# Using high-frequency phosphorus monitoring for water quality management: a case study of the upper River Itchen, UK

**DOI:** 10.1007/s10661-020-8138-0

**Published:** 2020-02-18

**Authors:** Gary R. Fones, Adil Bakir, Janina Gray, Lauren Mattingley, Nick Measham, Paul Knight, Michael J. Bowes, Richard Greenwood, Graham A. Mills

**Affiliations:** 1 School of the Environment, Geography and Geosciences, University of Portsmouth, Burnaby Road, Portsmouth, PO1 3QL UK; 2 Present Address: Cefas Laboratory, Pakefield Road, Lowestoft, Suffolk NR33 OHT UK; 3 Salmon & Trout Conservation, The Granary, Manor Farm, Burcombe Lane, Salisbury, SP2 0EJ UK; 4 Centre for Ecology & Hydrology, Maclean Building, Benson Lane, Crowmarsh Gifford, Wallingford, Oxfordshire OX10 8BB UK; 5 School of Biological Sciences, University of Portsmouth, King Henry I Street, Portsmouth, Hampshire PO1 2DY UK; 6 School of Pharmacy & Biomedical Sciences, University of Portsmouth, White Swan Road, Portsmouth, PO1 2DT UK

**Keywords:** Phosphorus, Nutrient, Chalk stream, Water sampling, River management

## Abstract

Increased concentrations of phosphorus (P) in riverine systems lead to eutrophication and can contribute to other environmental effects. Chalk rivers are known to be particularly sensitive to elevated P levels. We used high-frequency (daily) automatic water sampling at five distinct locations in the upper River Itchen (Hampshire, UK) between May 2016 and June 2017 to identify the main P species (including filterable reactive phosphorus, total filterable phosphorus, total phosphorus and total particulate phosphorus) present and how these varied temporally. Our filterable reactive phosphorus (considered the biologically available fraction) data were compared with the available Environment Agency total reactive phosphorus (TRP) values over the same sampling period. Over the trial, the profiles of the P fractions were complex; the major fraction was total particulate phosphorus with the mean percentage value ranging between 69 and 82% of the total P present. Sources were likely to be attributable to wash off from agricultural activities. At all sites, the FRP and Environment Agency TRP mean concentrations over the study were comparable. However, there were a number of extended time periods (1 to 2 weeks) where the mean FRP concentration (e.g. 0.62 mg L^−1^) exceeded the existing regulatory values (giving a poor ecological status) for this type of river. Often, these exceedances were missed by the limited regulatory monitoring procedures undertaken by the Environment Agency. There is evidence that these spikes of elevated concentrations of P may have a biological impact on benthic invertebrate (e.g. blue-winged olive mayfly) communities that exist in these ecologically sensitive chalk streams. Further research is required to assess the ecological impact of P and how this might have implications for the development of future environmental regulations.

**Electronic supplementary material:**

The online version of this article (10.1007/s10661-020-8138-0) contains supplementary material, which is available to authorized users.

## Introduction

Phosphorus (P) and nitrogen (N) are considered essential nutrient elements that are required by all living organisms for growth and energy transport (Hecky and Kilham 1988). P can often be the limiting nutrient for primary production in terrestrial and aquatic ecosystems (Elser 2012; Schindler et al. 2008; Vitousek et al. 2010). Elevated concentrations in the aquatic environment can lead to increased growth rate of algae and plants (Mainstone and Parr 2002), which over time can lead to eutrophication (Hilton et al. 2006; Withers et al. 2014). This can have adverse impacts on water quality, such as low oxygen concentrations, and for the characteristics of river habitats. These changes can cause undesirable disturbances to invertebrate and fish populations (UKTAG 2013).

Anthropogenic P can enter the aquatic environment through multiple routes, from either point or diffuse sources. Point sources include effluent from wastewater treatment plants (WWTP) (Jarvie et al. 2006), septic tanks (Zurawsky et al. 2004) and industrial discharges (Richards et al. 2015); diffuse sources are usually associated with overland and through flow from agricultural land (Macintosh et al. 2018) and contaminated groundwater (Nijboer et al. 2004). The average P concentration in European rivers has decreased markedly over the last two decades, with a 2.1% decrease per year (Fig. S1). This decrease in the concentration of P reflects improvements in wastewater treatment processes and the reduction of the P content in detergents (Foy 2007). However, national and regional variations persist in terms of P concentrations in the aquatic environment, and limiting P concentrations have not yet been achieved in most cases.

In the natural environment, P can exist in a variety of forms, including dissolved and particulate fractions. The dissolved form is an operationally defined fraction, for example that fraction that passes through a 0.20- or 0.45-μm membrane filter (Gimbert et al. 2007). The dissolved fraction encompasses both inorganic (e.g. orthophosphates and condensed or polyphosphates) and organic forms of P (e.g. nucleic acids, phosphoamides, phospholipids, proteins and sugar phosphates). Similarly, particulate P is that fraction that is retained on a 0.20- or 0.45-μm filter (Gimbert et al. 2007). This fraction can include clay and silt-associated organic and inorganic P, precipitates of authigenic origin and P-containing biological matter (Worsfold et al. 2016). Of these various fractions, dissolved inorganic P, in the form of orthophosphate, is the most important bioavailable fraction, as this can easily be incorporated by primary producers (Maruo et al. 2016). However, other some dissolved organic P species may also be utilised (Monbet et al. 2009; Sanudo-Wilhelmy 2006). The various operationally defined P fractions in natural waters, based on filtration and/or digestion, together with examples of the types of phosphorus species found in these fractions, have been reviewed by Worsfold et al. (2016).

The adverse effects of P in the aquatic environment have led to this substance being included in a number of national and international legislative frameworks and guidelines (e.g. the European Union’s Water Framework Directive (EU WFD 2000). Here, the WFD categorises natural waters as “high”, “good”, “poor” or “bad” quality or status with regard to phosphorus. In the UK, additional legislation related to P had been formulated by the UK Technical Advisory Group (UKTAG) on the Water Framework Directive. Revised standards proposed in their 2013 report (UKTAG 2013) adopted a new approach to setting P standards by taking into account the alkalinity and altitude of the specific field site.

Reliable monitoring of the different P species found in natural waters is a prerequisite to fulfil these legislative guidelines for P (Bowes et al. 2015). For this purpose, generally, low-volume discrete samples (bottle, grab or spot) are collected manually at a given time interval (e.g. monthly). However, this limited monitoring strategy fails to take into account any temporal variability in the concentration of P within a given water body (Bowes et al. 2009). Alternatively, automatic water samplers can be used for time series acquisition (e.g. hourly or daily) (Burke et al. 2002). This approach has provided insights into temporal nutrient dynamics on the time-scales of hydrological responses in agriculturally dominated catchments (Bieroza et al. 2014; Bieroza and Heathwaite 2015). Other methods have also been used, including sensors and autoanalysers (Clinton-Bailey et al. 2017; Rode et al. 2016; Wade et al. 2012) that can continually measure P concentrations in the field. This approach avoids issues of sample instability and allows very high frequency monitoring (sub-hourly) at low running costs; but these instruments and associated infrastructure are expensive and sites where they can be deployed are limited by the need for mains power supply. Other approaches include the use of passive samplers, such as the diffusive gradients in thin-film (DGT) technique (Mohr et al. 2015) or the Chemcatcher® (Knutsson et al. 2013). These samplers can be deployed in the field for extended periods, typically, weeks to yield time-weighted average concentrations. As both these devices use a diffusive barrier, they can only sequester the dissolved fraction of P.

The various monitoring procedures need to be used in conjunction with robust analytical techniques. The most commonly used technique is the “molybdenum blue” method combined with spectrophotometric detection, which determines molybdate-reactive orthophosphate (Nagul et al. 2015). Samples can be analysed using either a batch or flow-based approach. When using this method, all fractions of P need to be converted (e.g. by acid or alkaline digestion, or ultra-violet photo-oxidation) (Maher et al. 2002) to the detectable orthophosphate form. Other analytical methods include inductively coupled plasma methods with either atomic emission or mass spectrometric detection (Van Moorleghem et al. 2011).

There has been a recent interest in understanding the effects of elevated phosphorus concentrations on riverine benthic invertebrates (Everall et al. 2018). Numbers of organisms have declined in several UK rivers over the past 20 years, particularly in chalk streams (Bennett and Gilchrist 2010). Certain types of larvae (e.g. the blue-winged olive, *Serratella ignita*) are known to be sensitive to both fine sediment loading and elevated P concentrations (Larsen et al. 2011; Minutoli et al. 2013). A decrease in available benthic invertebrates can result in a decrease in the fish population (Salmon and Trout Conservation 2019). Recent ecological monitoring undertaken by the Salmon & Trout Conservation (S&TC) has shown that the upper River Itchen (Hampshire, UK) is in a poor ecological state compared with historical data and with accepted chalk stream parameters (Salmon and Trout Conservation 2019). It is suspected that recent increased P concentrations found in the river maybe responsible for this environmental degradation.

The aim of this study was to undertake high-frequency monitoring using automated water samplers over an extended period at several locations in the upper River Itchen that were known to be impacted by different P inputs. Using this approach, it was hoped that we could identify the main P species and how these varied temporally within this sensitive environment. Higher frequency sampling would also permit the identification of the specific time periods when any breaches of the UKTAG thresholds of P for this chalk stream environment occurred.

## Materials and methods

### Study area

**Fig. 1:**
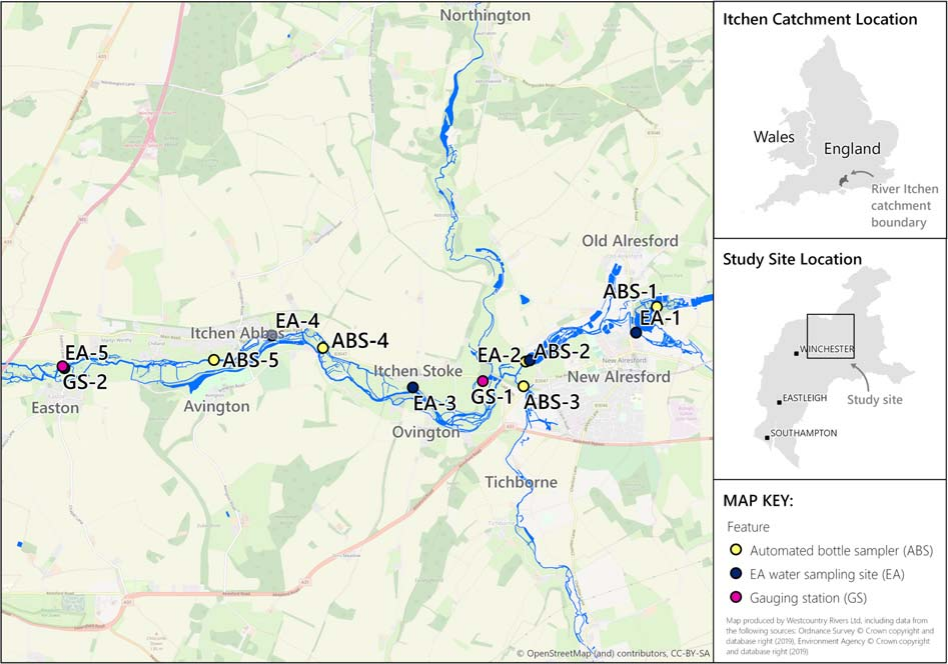
Map showing the location of the deployment sites of the five automated bottle samplers (ABS 1–5), the five Environment Agency water sampling sites (EA 1–5) and the two UK river flow gauging stations (GS 1–2) on the upper River Itchen

The Itchen is a river in Hampshire, southern England, UK (Fig. 1), and is 45 km long with a 400 km^2^ catchment area and having a mean discharge of 5.3 m^3^ s^−1^. The River Itchen is fed by three tributaries in its upper reaches; the Candover Stream, River Alre and the Cheriton Stream. The river flows through many Hampshire villages before entering the city of Winchester, from where it heads south, through a series of water meadows, before reaching the northern suburbs of Southampton. The Itchen is a typical chalk stream (hard, alkaline water) with a greater uniformity in physical characteristics along its entire length than other rivers of this type. As the river is spring fed, there is a relatively narrow range of seasonal variation in physical and chemical characteristics (e.g. water temperature is relatively constant), with a stable flow base flow index ~ 0.95 (Marsh and Hannaford 2008). The Itchen is designated as a site of Special Scientific Interest (SSSI) under the Wildlife and Countryside Act 1981 and an international Special Area of Conservation (SAC). It is considered one of the best locations for game fishing in the UK. There are several substantial abstractions for public water supply. The river supports a number of activities including fish farming and growing of watercress at the commercial scale. There are both agricultural run-off and domestic (waste water treatment and septic tanks) effluent inputs along the course of the river. These different anthropogenic pressures have resulted in the river failing to achieve SAC conservation targets (WWF-UK 2014).

### Field trial

Automatic bottle samplers (ABS) (Teledyne ISCO 3700–RS Hydro Ltd., Bromsgrove, UK) (Fig. S2 and Fig. S3) were deployed at five sites (ABS 1–5) on the upper River Itchen catchment between 27/05/2016 and 30/06/2017 (Fig. 1, Table S1 and Fig. S4).

Sampling sites ABS 1 and ABS 2 were located on the River Alre tributary above and below Old Alresford Pond. ABS 3 was located on a small tributary of the River Itchen (downstream of Alresford) before the confluence with the River Alre near Borough Bridge. ABS 4 and ABS 5 were located on the River Itchen, upstream (ABS 4) and downstream (ABS 5) of a trout fishery and a WWTP. Over the field trial locations along the river, there were also five sites where the Environment Agency take spot samples of water (EA 1–5) at irregular periods to measure phosphorus for regulatory purposes. In addition, there were two UK river flow gauging stations (GS 1–2) (see Fig. 1, Table S1 and Fig. S4).

### Automatic water sampling

Each ABS contained 24 polypropylene bottles (1 L) that were pre-cleaned (5% Decon for 24 h and rinsed with Milli-Q (Millipore) ultra-pure water). Mercuric chloride (2.5 mL of a 4 g L^−1^ solution) was added to each bottle as a preservative (Jarvie et al. 2002; Kattner 1999). The ABS were set to collect river water (500 mL) once every 24 h. Bottles were collected and replaced after every 24-day period. The water samples were then transferred to the laboratory and stored in the dark until analysis. The battery pack was changed every 48 days and the internal and external plastic sampling tubes were replaced every 6 months.

### Analysis of phosphorus

Analysis was carried out at the University of Portsmouth in their accredited (ISO 9001:2015) Environmental Chemistry Analytical Laboratory. All collected water samples were analysed within a week of their return to the laboratory. Three different fractions of phosphorus (filterable reactive phosphorus (FRP), total filterable phosphorus (TFP) and total phosphorus (TP)) were analysed. Total particulate phosphorus (TPP) was calculated as the difference between TP and TFP concentrations (Fig. S5). For the measurement of FRP and TFP, water samples (50 mL) were filtered (grade GF/F, (< 0.70 μm), 25-mm-diameter filters, Fisher Scientific Ltd., UK) and stored at 4 °C until analysis. For the measurement of TP (unfiltered) and TFP (filtered), water samples (20 mL) were digested (121 °C for 45 min) in an autoclave using a standard alkaline persulfate method (Koroleff 1983). The three phosphorus fractions were then analysed using a segmented continuous flow auto-analyser (SEAL Analytical QuAAtro (SEAL Analytical, Southampton, UK)) (Fig. S6). The auto-analyser used a phospho-molybdenum blue colourimetry method (Murphy and Riley 1962), with the reduced blue phospho-molybdenum complex read at 880 nm (Eisenreich et al. 1975). Calibration standards were prepared from potassium dihydrogen phosphate (Fisher Scientific, Loughborough, UK) covering the concentration range 0–124 μg L^−1^. Samples falling outside of this calibration range were diluted with Milli-Q water and then reanalysed. Phosphorus was quantified using an external calibration method using the instrument software (AACE, SEAL Analytical). The limit of detection was 0.31 μg L^−1^. Two external quality control standards (10 and 100 μg L^−1^) were run with each batch of twenty-four samples.

### Environment Agency sampling and analysis procedures

The regional Environment Agency collects in-frequent spot samples of water for the statutory monitoring of phosphorus. The Environment Agency measure a fraction which they refer to as orthophosphate, (total reactive form), referred here as total reactive phosphorus (EA-TRP). This fraction is obtained by allowing the particulate material in the water sample to settle over a period of time and then the supernatant decanted for analysis. EA-TRP was measured using the reaction with ammonium molybdate and antimony potassium tartrate under acidic conditions to form a complex which, when reduced with ascorbic acid, produces an intense blue colour. Absorbance was measured (880 nm) using a Konelab Discrete Analyser (Thermo Fisher Scientific, Hemel Hempstead, UK). EA-TRP data were obtained from the Environment Agency on-line water quality archive (https://environment.data.gov.uk/water-quality/view/landing).

### Discharge, flux and precipitation

Discharge data measured at the river flow gauging stations (GS 1–2) was obtained from the National River Flow Archive (https://nrfa.ceh.ac.uk/). Flux of FRP (kg day^−1^) was calculated from these values. Daily precipitation (mm) in the South East England area over the field trial period was obtained from HadUKP (http://www.metoffice.gov.uk/hadobs/hadukp/).

## Results and discussion

The five sites where the ABS (1–5) (Fig. 1 and Fig. S4) were located needed to be secure (e.g. avoiding areas of public access) and reachable for the whole period of the extended field trial. Hence, this limited the choice of locations available along the upper River Itchen. The different sites were selected to reflect the likely different anthropogenic inputs (e.g. agriculture, aquaculture, commercial growing of watercress and WWTP effluent) of P along the upper course of the river. Over the field trial locations, there were two river flow gauging stations (GS 1–2) where flow data could be gathered. These locations were not necessarily where the ABS were deployed. For the purpose of this study, GS 1 was used in association with ABS 1–4 and GS 2 was used in association with ABS 5. As these gauging stations were not exactly at the same location of the ABS, there was likely to be some discrepancies in the flow rates used to estimate the fluxes of P. Unlike in many rivers, the variation in flow over the year in the upper River Itchen is relatively low as it is spring water fed. For example, at GS 1, the river discharge varied only between 0.16 and 0.76 m^3^ s^−1^. The mean river discharge was higher at GS 2 (3.41 m^3^ s^−1^) compared with GS 1 (0.54 m^3^ s^−1^) due to more tributaries of the River Itchen being combined by the lower station. Over the trial, there were no periods where the river dried out. Daily precipitation data were only readily available for an area covering the South East of England. Hence, there could have been some local variations for rainfall received over the field trial at the specific sampling locations. Over the trial, there were many sporadic periods of increased precipitation (e.g. 22nd June 2016 up to ~ 34 mm); some were associated with an increase in river discharge. Over the trial period, there were 400 discrete automatic bottle samples available for collection. Due to operational reasons (i.e. blocked sampling tubes due to icing or battery failure), few samples were lost and, hence, were not available for analysis (Table 1).

**Table 1:** Mean (SD, maximum and minimum) concentration (mg L^−1^) of the various phosphorus fractions in the water samples collected by the automatic bottle samplers (ABS 1–5) over the deployment period 27/05/16–30/06/17. Environment Agency (EA) data for the mean (SD, maximum and minimum) concentration of phosphorus (mg L^−1^) from their nearest sampling site is also given

Site number	Phosphorus fraction	Number of samples (n)	Mean	SD	Max	Min	Nearest equivalent EA Site	Phosphorus fraction	Number of samples (n)	Mean	SD	Max	Min
			mg L^−1^							mg L^−1^			
ABS 1	FRP	349	0.041	0.075	0.695	< LOD	EA 1 (Old Alresford Pond)	EA-TRP	4	0.051	0.027	0.087	0.022
	TFP	349	0.067	0.110	1.134	0.003							
	TP	349	0.405	0.496	3.792	0.022							
	TPP	349	0.338	0.444	3.493	0.006							
ABS 2	FRP	392	0.043	0.104	0.714	< LOD	EA 2 (River Arle at Drove Lane)	EA-TRP	4	0.035	0.015	0.052	0.016
	TFP	392	0.078	0.140	0.886	0.001							
	TP	392	0.362	0.601	3.043	0.003							
	TPP	392	0.284	0.491	3.034	0.001							
ABS 3	FRP	389	0.016	0.022	0.236	< LOD	No EA data for this site						
	TFP	389	0.037	0.040	0.312	< LOD							
	TP	389	0.166	0.131	0.830	0.004							
	TPP	389	0.129	0.114	0.704	0.004							
ABS 4	FRP	392	0.020	0.042	0.339	< LOD	EA 3 (River Itchen at Itchen Stoke)	EA-TRP	4	0.021	0.005	0.027	0.016
	TFP	392	0.048	0.055	0.773	0.003							
	TP	392	0.193	0.248	2.414	0.031							
	TPP	392	0.148	0.211	2.031	0.001							
ABS 5	FRP	394	0.053	0.096	0.527	0.001	EA 5 (River Itchen at Easton)	EA-TRP	9	0.033	0.012	0.053	0.012
	TFP	394	0.098	0.164	1.357	0.007							
	TP	394	0.613	0.644	3.528	0.036							
	TPP	394	0.514	0.537	2.640	0.018							

FRP, filterable reactive phosphorus; TFP, total filterable phosphorus; TP, total phosphorus; TPP, total particulate phosphorus; EA-TRP, Environment Agency total reactive phosphorus; LOD, limit of detection

### Variation in concentration of phosphorus fractions

**Fig. 2:**
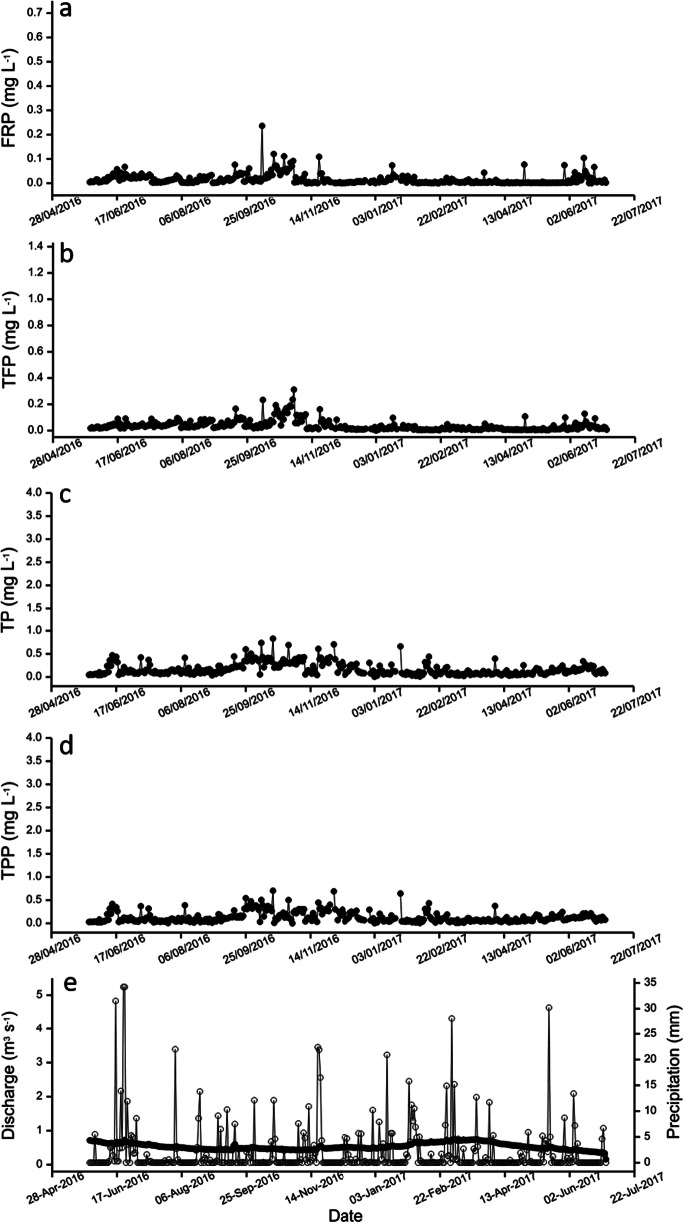
Variation in the concentration of phosphorus (mg L^−1^) fractions (•) in water samples collected (27 May 2016–30 June 2017) at site ABS 3 on the upper River Itchen **a** filterable reactive phosphorus (FRP); **b** total filterable phosphorus (TFP); **c** total phosphorus (TP); **d** total particulate phosphorus (TPP). Precipitation (mm) (**○**) and river discharge (m^3^ s^−1^) (•) measured at gauging station GS 1 over this period is shown **e**

**Fig. 3:**
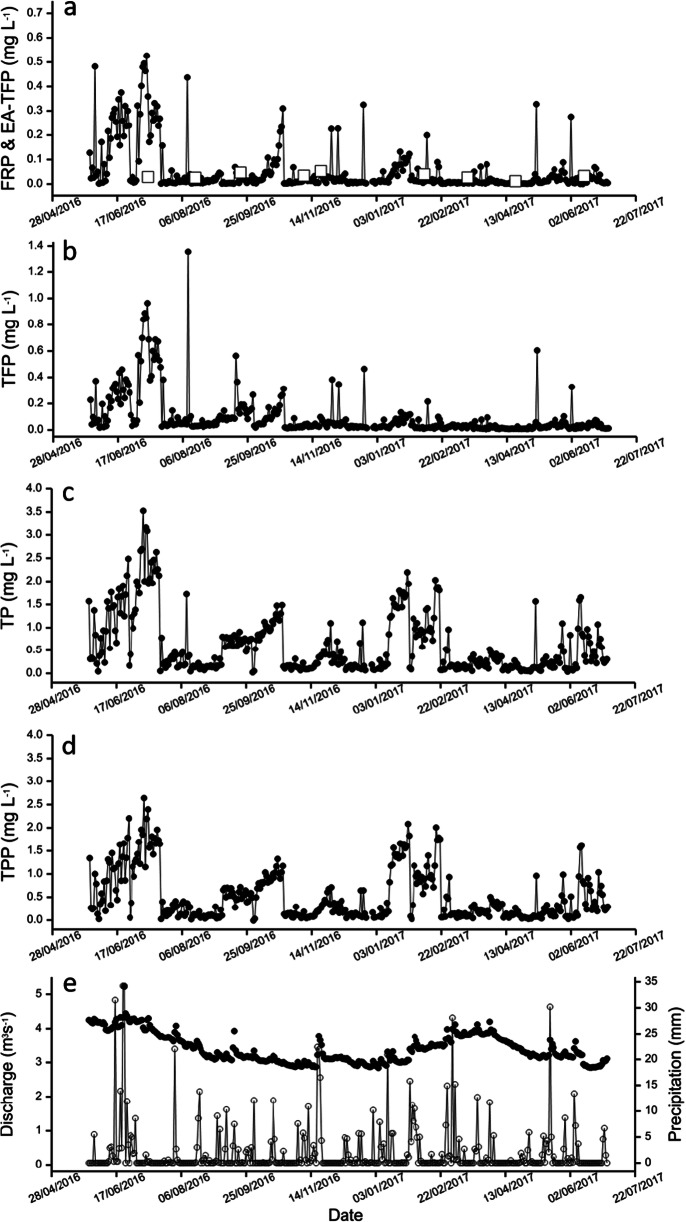
Variation in the concentration of phosphorus (mg L^−1^) fractions (•) in water samples collected (27 May 2016–30 June 2017) at site ABS 5 on the upper River Itchen **a** filterable reactive phosphorus (FRP); **b** total filterable phosphorus (TFP); **c** total phosphorus (TP); **d** total particulate phosphorus (TPP). Precipitation (mm) (**○**) and river discharge (m^3^ s^−1^) (•) measured at gauging station GS 1 over this period is shown **e**. The concentration of total reactive phosphorus (mg L^−1^) (EA-TRP) (▯) measured by the Environment Agency at EA 5 (River Itchen at Easton) is shown in **a**

The variation in the concentration of P in the four fractions (FRP, TFP, TP and TPP) measured over the field trial is shown in Figs. 2 and 3 for sites 3 and 5. Corresponding data for sites 1, 2 and 4 are shown in Figs. S7–S9. To aid interpretation, the concentration of P for each of the fractions found at the five sites is plotted on the same scale in each of the figures. Composite plots showing the variation of the four P fractions at each site are shown in Figs. S10–S14. The upper River Itchen has the potential for a number of different inputs of P and this adds complexity to understanding the dynamics of the riverine system. Hence, the concentration of P was highly variable during the trial period. Data for the mean, maximum and minimum concentration of the various P fractions measured is shown in Table 1.

ABS 1 was located on the Alre tributary downstream of a watercress-growing facility and upstream of Old Alresford Pond. At this site, TPP generally dominated and on occasions accounted for up to 98% (mean = 79%) of the TP present (Fig. S7 and Fig. S10). Increases in TPP were associated with precipitation events likely to cause soils from agricultural land where P-based fertilisers have been used to be washed off into the river (Jarvie et al. 2013; Withers et al. 2014). Other agricultural sources of P include fertilisers, livestock faeces and vegetation (Hodgkinson and Withers 2007). Another potential cause of the increase in TPP is from the resuspension of P-rich bed sediments that have been sequestered from the water column under low flow conditions (Bowes and House 2001; Bowes et al. 2005). However, chalk rivers typically have low suspended solids, but can have high and sporadic suspended solid concentrations due to specific anthropogenic activities, e.g. from cleaning watercress production beds (Casey and Smith 1994). P associated with the particulate fraction can become a secondary source for plant growth as under favourable conditions; it can become bioavailable (Stutter et al. 2010; Wang and Pant 2010). There was one notable increase in concentration of FRP, between 22nd and 27th of June 2016, accounting for up to 40% of TP. A potential source was P-based fertiliser being applied to the watercress beds during this peak growing period. The use of P per unit area of watercress bed is very high as plant uptake is inefficient (Cox 2009). This increase was not observed in the following year; however, data for FRP for the period 4th of June–21st of June 2017 were not available.

ABS 2 was sited below a large trout fishery. Again at this site, TPP dominated and on occasions accounted for up to 99% (mean = 69%) of the TP present (Fig. S8 and Fig. S11). There was evidence that increased flow events caused by sustained precipitation (e.g. late November 2016 and early February 2017) led to increased concentrations of TPP and FRP at this site. Fish farming activities at this site could contribute to the input of P. Most farms use feeds that are supplemented with P. The retention of P by fish is known to be variable being species dependant. Low assimilation of P leads to the release of enriched waste streams entering rivers (Lazzari and Baldisserotto 2008).

Site ABS 3 was selected as it was expected that this small tributary would have minimal inputs of P. Data shown in Fig. 2 and Fig. S12 confirmed this supposition. Low-level background sources that contribute to P concentrations could include soil weathering, riverbank erosion and riparian vegetation (Daldorph et al. 2015). At this site, TPP still dominated the fractionation profile and accounted for up to 99% (mean = 75%) of the TP present.

The fraction profile for ABS 4 is shown in (Fig. S9 and Fig. S13). Similarly to ABS 3, this was shown to be a low P impacted site as there were no known local point source inputs. Again, the profile was dominated by TPP. Over the field trial, there was one period (mid-May 2017) of increased P associated with increased precipitation.

Site ABS 5 received point source inputs from a trout fishery and a WWTP as well as other potential sources of P further up the catchment and had the highest TP concentrations observed in this study. The complex profile for the different fractions of P throughout the trial period (Fig. 3 and Fig. S14) made it challenging to assign events to any one input. However, domestic and industrial effluents discharged into surface waters from WWTP are known to be a major point source of P (Comber et al. 2012). Along this rural catchment, unplanned inputs from septic tank systems used for domestic waste disposal may have also contributed to the P concentrations at ABS 5 (Arnscheidt et al. 2007). Again, the profile was dominated by TPP where it accounted for up to 99% (mean = 82%) of the TP present.

### Comparison with regulatory monitoring

Over the trial period, the Environment Agency collected and analysed a number of spot water samples for P from their routine sampling stations (EA 1, EA 2, EA 3 EA 5) (Fig. 1). These were for regulatory and statutory purposes. For logistical and practical reasons, these stations, however, did not coincide precisely with the location of the ABS sites. The sites nearest to each other on the river have been used for comparative purposes (Table 1). It should be noted that the Environment Agency measure orthophosphate, known as total reactive phosphorus (EA-TRP). This fraction is similar to FRP in our analytical procedure. It was expected that the corresponding EA-TRP values would be slightly higher than our FRP values due to the potential for the inclusion of some particulate material in the Environment Agency analyses.

EA-TRP values for each station are plotted alongside the corresponding daily FRP values (except for ABS 3 as there was no Environment Agency sampling point near to this location) in Figs. 2 and 3, and in Figs. S7–S9. Considering the above caveats of the study, there was a broad agreement between the concentrations of P measured by the two monitoring methods. The mean concentrations of P obtained by both methods were likewise in good agreement (Table 1). In contrast, however, at each sampling point, there was approximately an order of magnitude difference in the maximum concentration (FRP > EA-TRP) and minimum concentration (FRP < EA-TRP) measured by these methods. Therefore, the limited frequency of the statutory monitoring undertaken by the Environment Agency does not capture the full range of P concentrations at each site and is unable to detect any sporadic pollution incidents. This agrees with previous studies where it has been shown that such rapid changes in the concentration of P can be missed unless ABS are used (Bowes et al. 2009; Johnes 2007). Hence, this could lead to a misrepresentation of water quality and associated ecological health of the river (Skeffington et al. 2015).

FRP and EA-TRP mean concentrations over the trial period can be compared with UKTAG P standards (Table S2) and other standards appertaining to SSSI habitats (Table S3). The part of the upper River Itchen in our study is classified as low altitude (< 80 m) and high alkalinity (> 50 mg L^−1^ CaCO_3_). Using the UKTAG criteria (UKTAG 2013) (Table S2), the five locations were classified as either high (≤ 0.036 mg L^−1^ P) or good (≤ 0.069 mg L^−1^ P) ecological status. Apart from ABS 5 (FRP = good status; EA-TRP = high status), there was good agreement between the classifications for both monitoring methods. It was also possible to make a comparison with the mean concentrations obtained by the two monitoring methods with the Common Standards Monitoring Guidance for Rivers (Table S3). Here, using flow data from the gauging stations, sites ABS 1–4 were classified as headwaters (target ≤ 0.04 mg L^−1^ P) and site ABS 5 as a river (target ≤ 0.05 mg L^−1^ P) (JNCC 2016). Overall, the upper parts of the river were generally consistent with favourable conditions of a SSSI/SAC riverine habitat.

Looking at the FRP concentrations from the five ABS, over the trial period, there were several extended periods where levels were in excess of these environmental standards. For example, at site ABS 2 (Fig. S8) between 6th and 12th of February 2017, there was a week-long period of elevated FRP (mean = 0.615 mg L^−1^). Similarly, at ABS 5 (Fig. 3), there were two periods (10th–26th June of 2016 and 3rd–20th of July 2016) where the FRP concentration was elevated (mean = 0.250 and 0.321 mg L^−1^ respectively). During these extended periods, using the UKTAG criteria (Table S2), the status of these parts of the river would be classified as poor. The precise environmental impact of these extended periods of elevated concentrations of FRP in a sensitive riverine environment is presently not fully understood.

In riverine environments, elevated long-term concentrations of P can lead to the proliferation of nuisance phytoplankton and both epiphytic and benthic algae (Azevedo et al. 2015). It is well known that nutrient enrichment can result in a reduction in macroinvertebrate community richness (Friberg et al. 2010). Nutrient enrichment mesocosm experiments in similar chalk stream environments (River Lambourn, Berkshire and River Frome, Dorset, southern England) have shown that periphyton growth is not limited by FRP concentration in excess of 0.1 mg L^−1^ (Bowes et al. 2007; McCall et al. 2017). Therefore, the regular peaks in FRP concentration above this concentration will relieve any phosphorus limitation in the Itchen.

Recently, Everall et al. 2018 investigated, in the laboratory, the effect of increased concentrations of orthophosphate (maximum exposure = 0.3 mg L^−1^) in the presence of increased suspended fine sediment (maximum level = 25 mg L^−1^) on the early life stages of the blue-winged olive mayfly (*Serratella ignita*). Exposures were either 72 or 183 days. Egg mortality in control treatments was around 6% compared with 45% in treatments with 25 mg L^−1^ suspended sediment and 52% in 0.3 mg L^−1^ orthophosphate exposures. Even relatively modest concentrations of orthophosphate (0.1 mg L^−1^) had effects on egg survival to hatching. These exposure values are similar to those mean concentrations found during the elevated periods as described above. It should be noted that these elevated periods were shorter than the mesocosm exposure experiments described by Everall et al. (2018). However, over the trial period in the upper River Itchen, there were many short- and long-term incidences where the concentration of FRP (taken as equivalent to orthophosphate in the Everall et al. study) exceeded 0.3 mg L^−1^ (see for example Fig. 3 and Fig. S8). The biological effects on benthic biota due to these cyclical increases in FRP above a high or good ecological status as defined by the UKTAG criteria are difficult to predict with certainty. Recent field studies (e.g. using kick test sampling) (Salmon and Trout Conservation 2019) along the River Itchen highlighted that there are fewer riverfly species present than would be expected in a healthy chalk stream. It is impossible to ascertain whether bioavailable P is directly responsible for these observations. The impact of orthophosphate on biota is likely to be complex and have multi-faceted synergistic and antagonistic interactions with other pollutants and environmental stressors that may be present in the aquatic environment and should be the subject of further research (Everall et al. (2018).

## Concentration–discharge relationships and fluxes

**Fig. 4:**
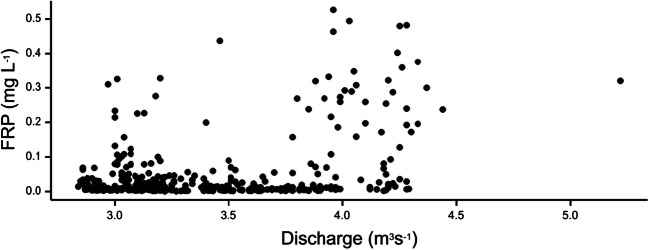
Variation in the concentration of filterable reactive phosphorus (FRP) (mg L^−1^) with river discharge (m^3^ s^−1^) at site ABS 5 over the deployment period (27 May 2016–30 June 2017). River discharge data obtained from gauging station 2 (Itchen at Easton, 42016)

The concentration of the different P fractions can be plotted against river discharge to ascertain if a relationship exists between these two variables, i.e. does the concentration decrease or increase with increasing flow. FRP (Fig. 4 and Figs. S15–18) and TP (Figs. S19-S23) concentration–discharge relationships for the five sampling sites (ABS 1–5) have been plotted. Discharge data for ABS 1–4 were obtained from GS 1 and data for ABS 5 from GS 2. As expected for this spring-fed chalk stream river, discharge values at the two gauging stations did not vary greatly over the trial period.

There was evidence for a limited increase in FRP with increasing discharge (≥ 0.7 m^3^ s^−1^) for two of the headwater sampling sites (ABS 1 and ABS 2) (Figs. S15 and S16). For the small tributary sampling site (ABS 3), there was no observable relationship between FRP concentrations and discharge (Fig. S17). At ABS 4, an increase in the concentration of FRP was associated with a lower discharge of 0.45 m^3^ s^−1^ (Fig. S18). At the lower river sampling point (ABS 5), much higher discharges were observed, with an increase in FRP associated with a discharge above 3.75 m^3^ s^−1^ (Fig. 4). Different profiles were obtained for TP. At sampling sites ABS 1 and ABS 2 (Figs. S19 and S20), there were two distinct regimes of elevated TP associated with discharges at ~ 0.5 and ~ 0.7 m^3^ s^−1^. For the tributary (ABS 3), there was a similar pattern; however, TP generally decreased with increasing discharge (Fig. S21). This pattern of decreasing TP with discharge was more evident at ABS 4 (Fig. S22). At ABS 5, a similar pattern was observed for TP as was found for FRP (Fig. S23).

Assigning these changes in profile of P to specific events occurring along the river is difficult as they may be multi-factorial, although some trends can be observed. For example, using site ABS 5 (Fig. 4 and Fig. S23), three different events appear to be occurring. At reduced discharge (~ 3 m^3^ s^−1^) the higher concentrations of P are likely to be associated with a constant point source input such as WWTP. As the discharge increases, the concentration of P decreases due to dilution effects. This has been observed previously by Bowes et al. (2009) on the River Frome (Dorset, UK). The higher discharges (≥ 3.75 m^3^ s^−1^) were associated with a series of rainfall events (14th of June–8th of July 2016) leading to an increase in P. The potential sources of P are likely to be from wash-off from field drains and other near-channel stored phosphorus sources (Bowes et al. 2015). There is also a contribution from the remobilisation of bed-sediment under these higher discharge regimes (Bowes and House 2001; Jarvie et al. 2012).

Mean daily flux (kg day^−1^) estimates for FRP and TP were calculated using the data obtained from the high frequency ABS (Table 2). Plots of FRP flux against time are shown in Figs. S24–S28. The higher fluxes found at the two upper sampling sites (ABS 1 and ABS 2) reflect inputs from anthropogenic activities on these stretches of the catchment. As expected, the flux of FRP and TP was at a maximum value at the furthest sampling point (ABS 5) down the catchment due to additional inputs of P (e.g. WWTP effluent) and increases in river discharge. Our calculated mean fluxes were in good agreement with previous values (2 to 171 kg day^−1^) measured between 1997 and 2002 for different stretches of the River Itchen (Cox 2009).

**Table 2:** Mean (SD, maximum and minimum) daily flux (kg day^−1^) of FRP and TP measured in the water samples collected by the automatic bottle samplers (ABS 1–5) over the deployment period 27th of May 2016–30th of June 2017

Site number	Number of samples (*n*)	FRP (kg day^−1^)				TP (kg day^−1^)			
		Mean	SD	Max	Min	Mean	SD	Max	Min
ABS 1	349	2	4	38	< 1	19	25	175	< 1
ABS 2	392	2	6	42	< 1	17	30	171	< 1
ABS 3	389	< 1	< 1	10	< 1	7	6	34	< 1
ABS 4	392	< 1	2	16	< 1	8	10	94	1
ABS 5	394	17	33	180	< 1	188	215	1228	10

## Conclusions

This work is one of the few studies that have investigated the impact of P in a sensitive chalk stream river that is now becoming recognised to have a poor ecology. This was affected by the use of daily water sampling at five strategic locations along the upper River Itchen over an extended sampling period. These data provide important information of the variation in concentrations of P species over an annual cycle. Critically, there were a number of extended time periods where the mean FRP concentration exceeded the existing regulatory values for this type of river. Often, these exceedances were missed by the limited regulatory monitoring procedures undertaken by the Environment Agency in this catchment. There is evidence that these spikes of elevated concentrations of P may have a biological impact on the benthic invertebrate communities. Further investigations into the biological impact of these spikes in P concentration are urgently required. The values and profiles found in this study could be used in laboratory mesocosm experiments where a range of biota is exposed at environmentally relevant concentrations over different exposure regimes. The impact of fine sediment in the form of suspended particulate matter also needs to be considered as this can be an additional source of P. We have shown that only by the use of high frequency monitoring can we begin to understand the complexity of P within chalk stream habitats.

## Electronic supplementary material

ESM 1 (DOCX 10982 kb)
