# Supplementary material for: Using high-frequency phosphorus monitoring for water quality management: a case study of the upper River Itchen, UK
Source: Environ Monit Assess. 2020 Feb 18;192(3):184. doi: 10.1007/s10661-020-8138-0 (PMC7028801; doi:10.1007/s10661-020-8138-0)
Supplement: Supplementary file 1 — (DOCX 10982 kb) [file 10661_2020_8138_MOESM1_ESM.docx]

Supporting information

**Use of high-frequency phosphorus monitoring for water quality management: a case study of the upper River Itchen, UK**

Gary R. Fones^a*^, Adil Bakir^a†^, Janina Gray^b^, Lauren Mattingley^b^, Nick Measham^b^, Paul Knight^b^, Michael J. Bowes^c^, Richard Greenwood^d^ and Graham A. Mills^e^

^a^School of Earth & Environmental Sciences, University of Portsmouth, Burnaby Road, Portsmouth, PO1 3QL, UK

^b^Salmon & Trout Conservation, The Granary, Manor Farm, Burcombe Lane, Salisbury, SP2 0EJ, UK

^c^Centre for Ecology & Hydrology, Maclean Building, Benson Lane, Crowmarsh Gifford, Wallingford, Oxfordshire, OX10 8BB, UK

^d^School of Biological Sciences, University of Portsmouth, King Henry I Street, Portsmouth, Hampshire, PO1 2DY, UK

^e^School of Pharmacy & Biomedical Sciences, University of Portsmouth, White Swan Road, Portsmouth, PO1 2DT, UK

*Corresponding author: gary.fones @port.ac.uk; Tel: 44(0) 2392 842 252

^†^Present address: Cefas Laboratory, Pakefield Road, Lowestoft, Suffolk, NR33 OHT, UK

**
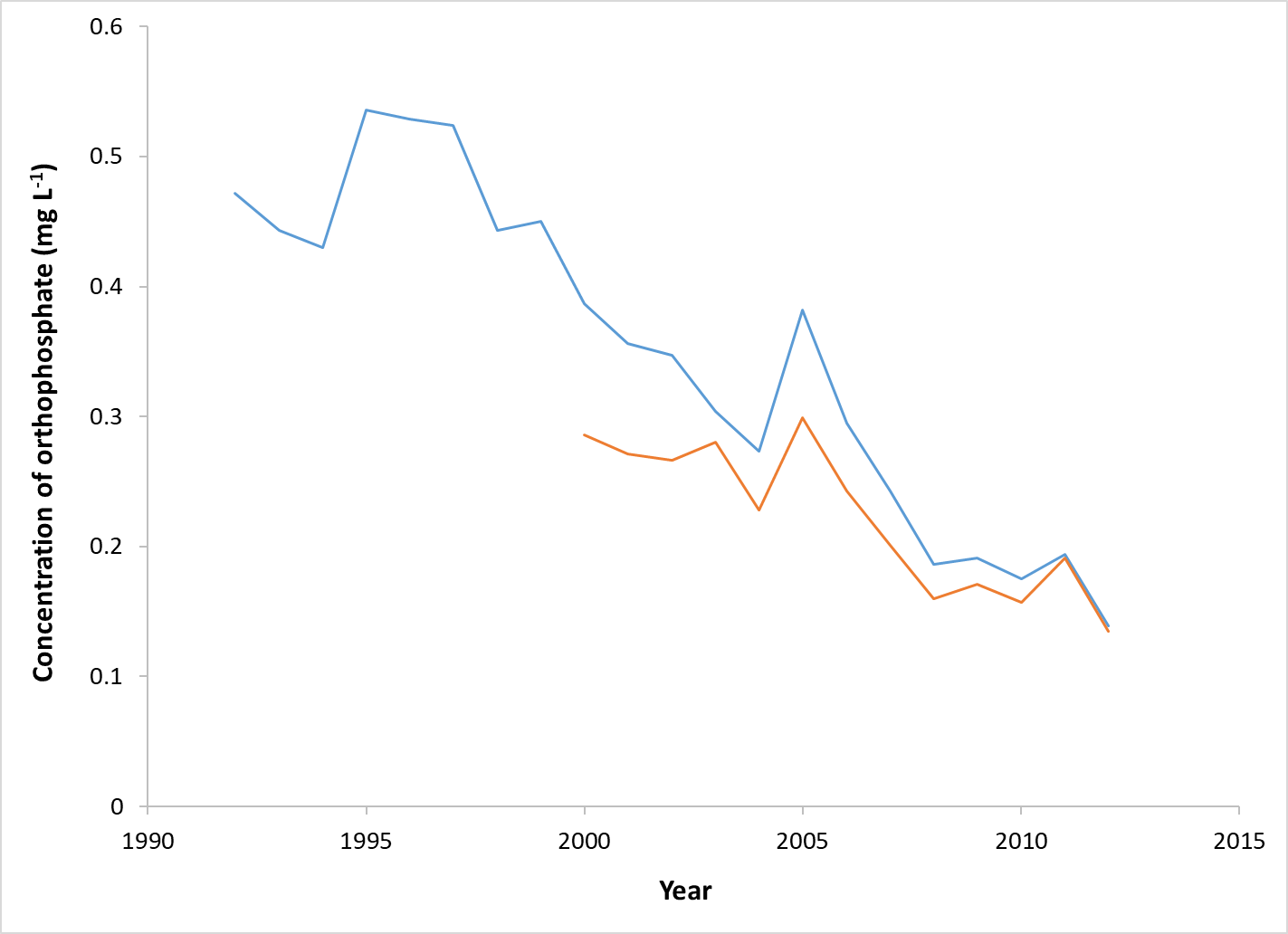
**

**Figure S1.** Average annual mean concentration of orthophosphate (mg L^-1^) measured at river stations in the United Kingdom between 1992 and 2012. Data for 1992 – 2012 was collected from 70 river stations and data for 2000 – 2012 was collected from 155 river stations (European Environment Agency, 2017; <https://www.eea.europa.eu/data-and-maps/indicators/nutrients-in-freshwater/nutrients-in-freshwater-assessment-published-6>).

**Table S1.** Location of the deployment sites for the automated bottle samplers, Environment Agency (EA) water sampling sites and UK river flow gauging stations on the upper River Itchen. Locations are numbered in the direction of river flow.

| **Description** | **Grid Reference** | **Latitude** | **Longitude** |
| --- | --- | --- | --- |
| Automated bottle sampler 1 | SU 59159 33300 | 51.095965 | -1.1565661 |
| Automated bottle sampler 2 | SU 57382 32564 | 51.089527 | -1.1820562 |
| Automated bottle sampler 3 | SU 57352 32229 | 51.086518 | -1.1825376 |
| Automated bottle sampler 4 | SU 54631 32750 | 51.091468 | -1.2213039 |
| Automated bottle sampler 5 | SU 53153 32587 | 51.090141 | -1.2424301 |
|  |  |  |  |
| EA water sampling site 1  (Old Alresford Pond) | SU 58878 32951 | 51.092852 | -1.1606420 |
| EA water sampling site 2  (River Alre Drove Lane) | SU 57435 32581 | 51.089671 | -1.1812900 |
| EA water sampling site 3  (River Itchen at Itchen Stoke) | SU 55850 32211 | 51.086501 | -1.2039760 |
| EA water sampling site 4  (Itchen Abbas Trout Farm Inlet) | SU 53938 32911 | 51.092977 | -1.2311810 |
| EA water sampling site 5  (River Itchen at Easton) | SU 51127 32474 | 51.089305 | -1.2713790 |
|  |  |  |  |
| Gauging station 1  (Candover Stream at Borough Bridge: 42009) | SU 56800 32300 | 51.087211 | -1.1904067 |
| Gauging station 2  (Itchen at Easton: 42016) | SU 51100 32500 | 51.089545 | -1.2717537 |

**River gauging station 1: Candover Stream at Borough Bridge, EA Southern (42009)**

Station: Crump profile weir (crest: 2.99 m broad). Modular throughout the range. All flows contained and no bypassing. Monthly gaugings available from 1956. Run-off reduced by surface ground water abstractions but augmentation of low flows is important in notable droughts (e.g. 1976, 1997 and 2005). Cress-bed management may produce hydrograph spikes. The ground water and topographical divides differ considerably. Catchment: An unresponsive catchment (chalk with significant drift cover). Many perennial springs - often supporting cress beds. Predominantly rural land use with some woodland.

**River gauging station 2: Itchen at Easton, EA Southern (42016)**

Station: Electromagnetic gauging station with insulated bed. Largely natural, base-flow dominated, regime but ground water augmentation during severe droughts. Catchment: A predominantly Chalk catchment with significant patches of superficial deposits. Largely rural with some woodland and scattered settlements.

**
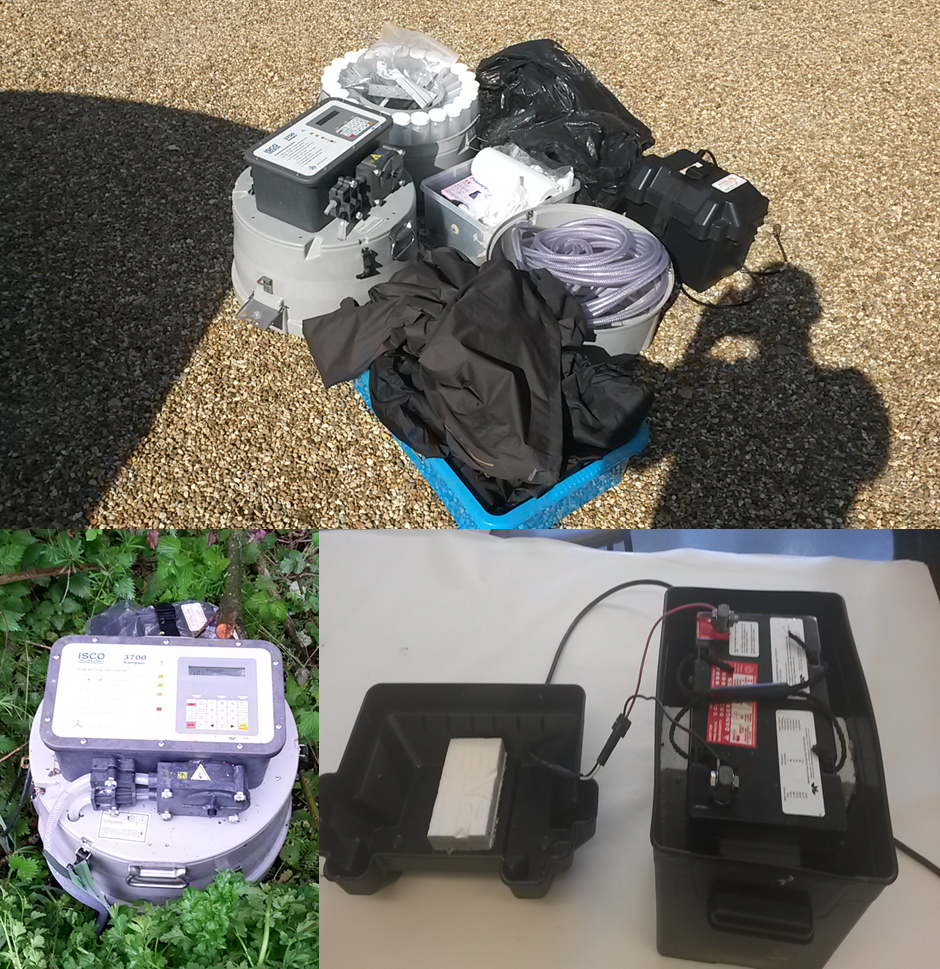
**

**Figure S2.** Component parts of the automatic bottle sampler (model Teledyne ISCO 3700).


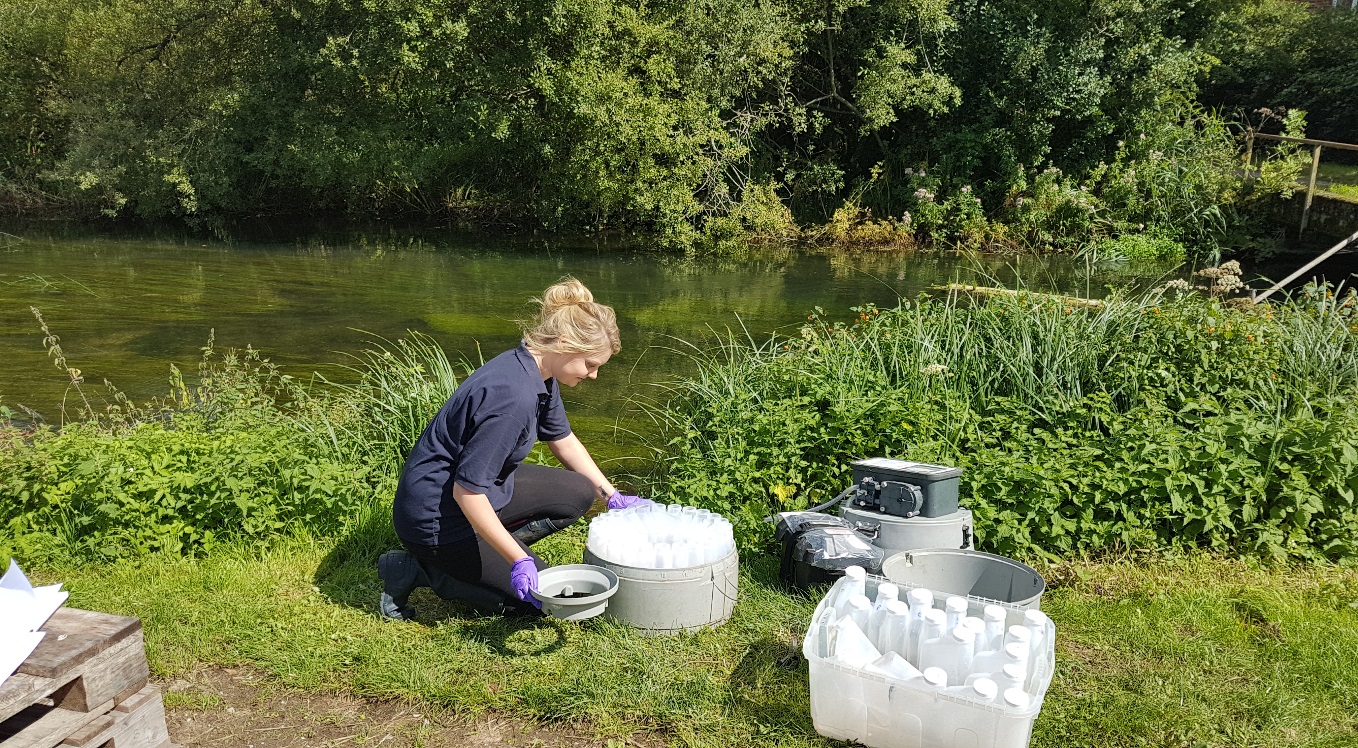


**Figure S3.** Collection and removal of the sample bottles from the automated bottle sampler at a riverine site.

**
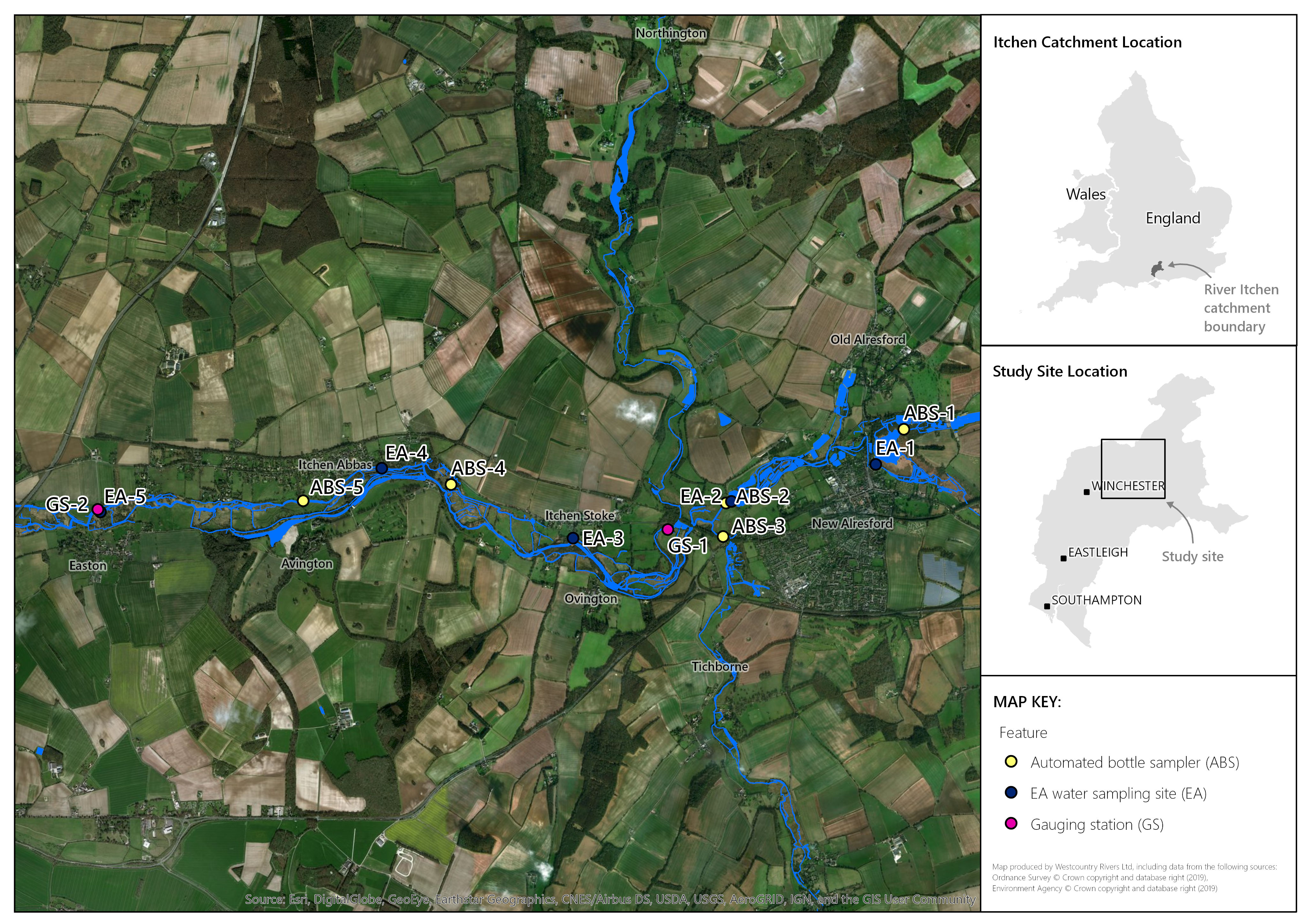
**

**Figure S4.** Satellite land use map showing the location of the deployment sites of the five automated bottle samplers (ABS 1-5), the five Environment Agency water sampling sites (EA 1-5) and the two UK river flow gauging stations (GS 1-2) on the upper River Itchen.

**
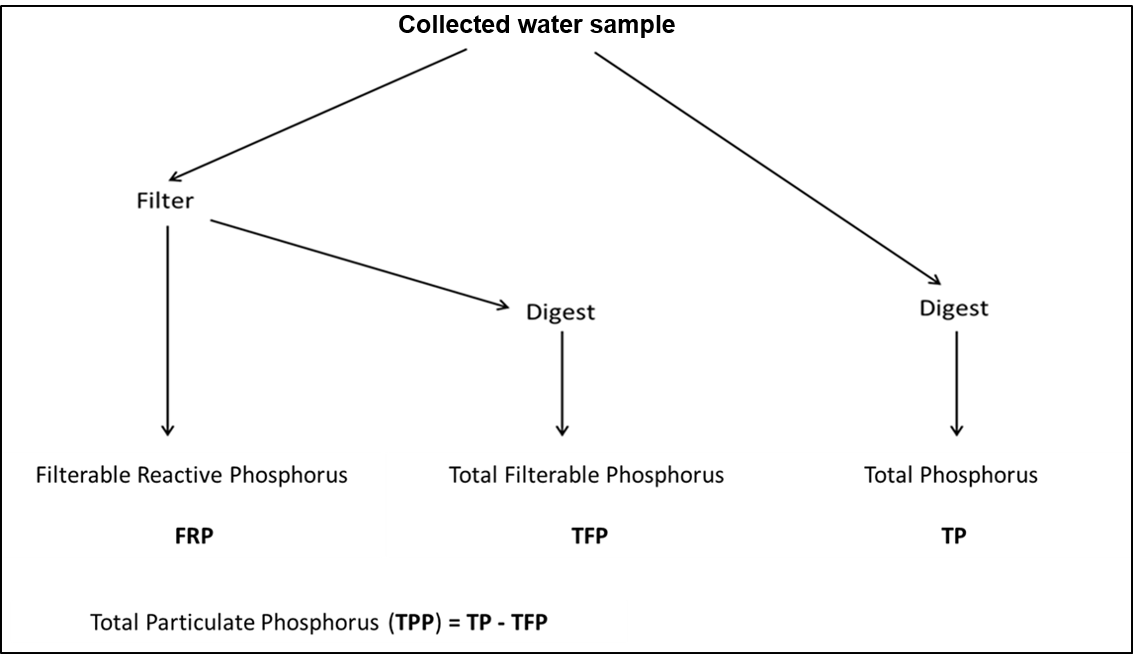
**

**Figure S5.** The different fractions of phosphorus as defined in this study.

**
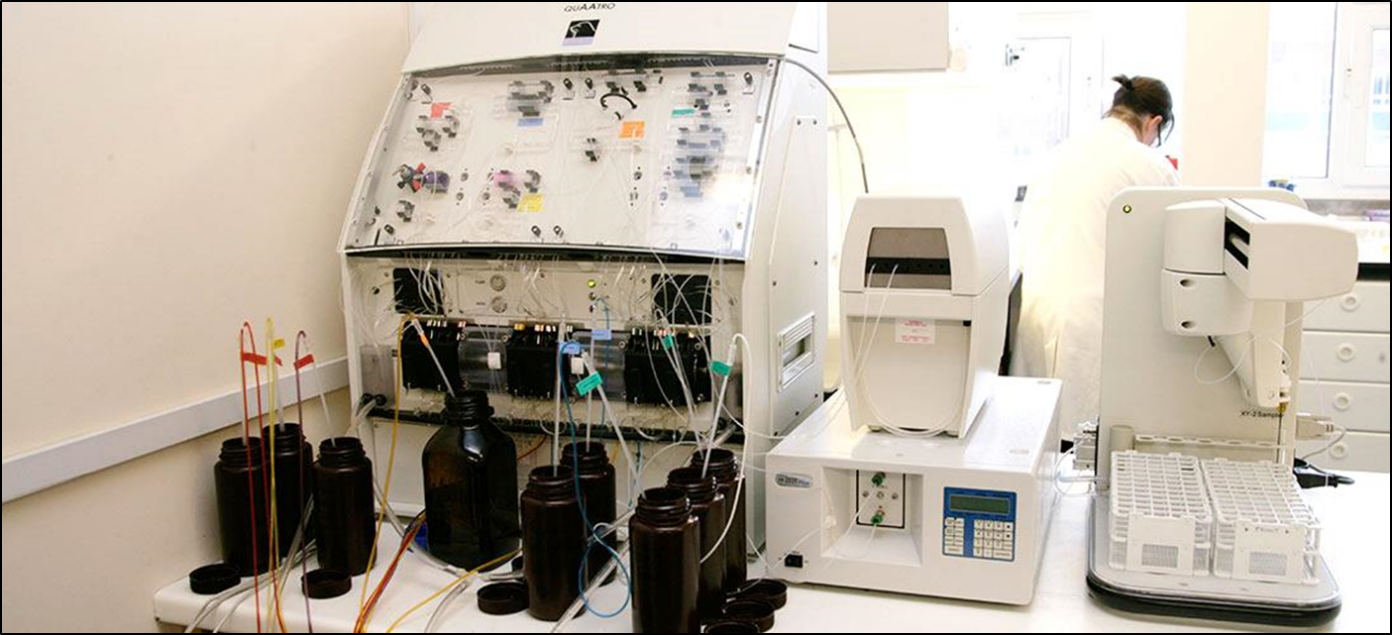
**

**Figure S6.** SEAL Analytical QuAAtro nutrient auto-analyser with auto-sampler.

**
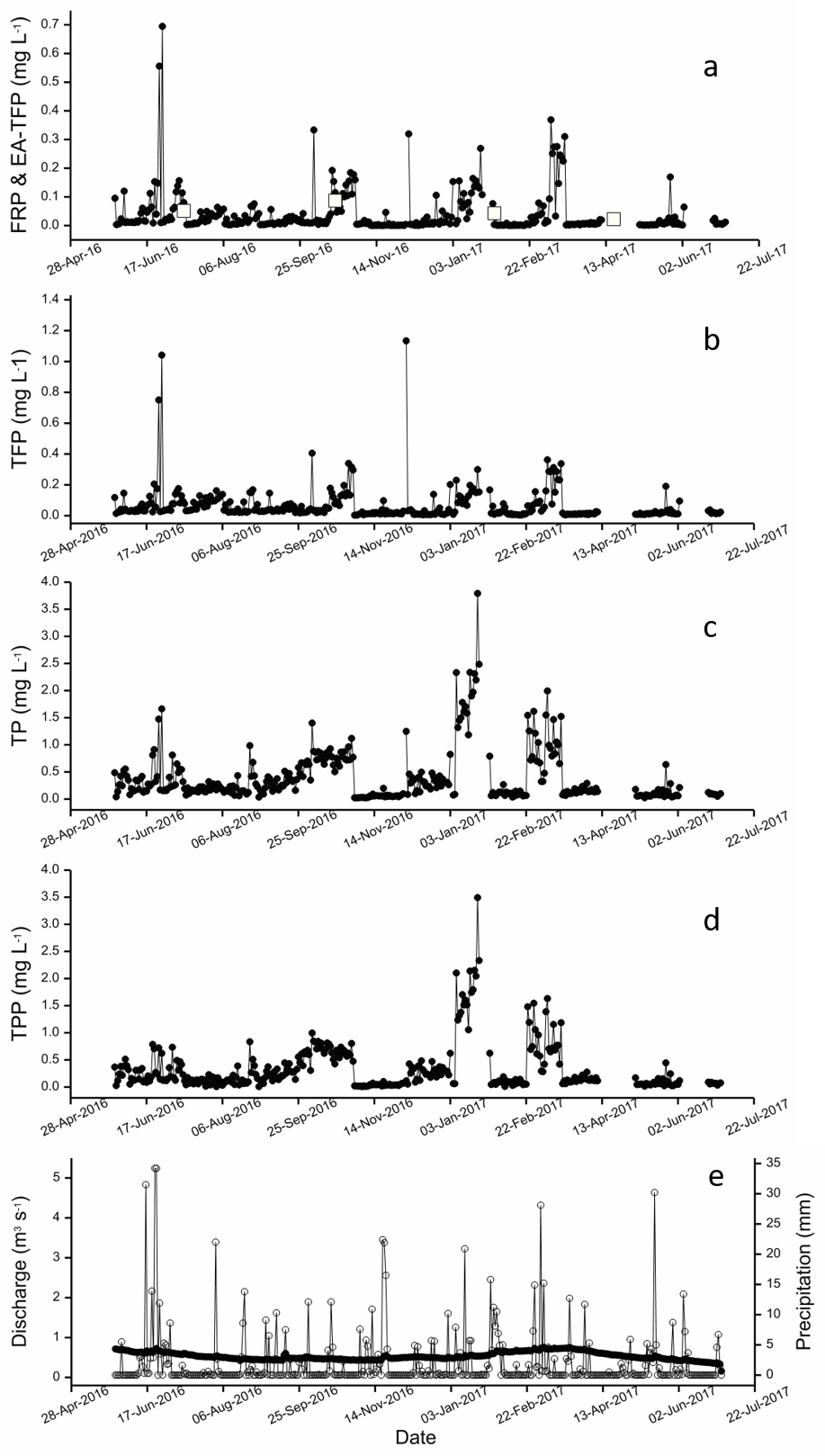
**

**Figure S7.** Variation in the concentration of phosphorus (mg L^-1^) fractions (•) in water samples collected (27 May 2016 – 30 June 2017) at site ABS 1 on the upper River Itchen (a) filterable reactive phosphorous (FRP); (b) total filterable phosphorous (TFP); (c) total phosphorous (TP); (d) total particulate phosphorous (TPP). Precipitation (mm) (⭘) and river discharge (m^3^ s^-1^) (•) measured at gauging station GS 1 over this period is shown (e). The concentration of total reactive phosphorous (mg L^-1^) (EA-TRP) () measured by the Environment Agency at EA 1 (Old Alresford Pond) is shown in (a).

**
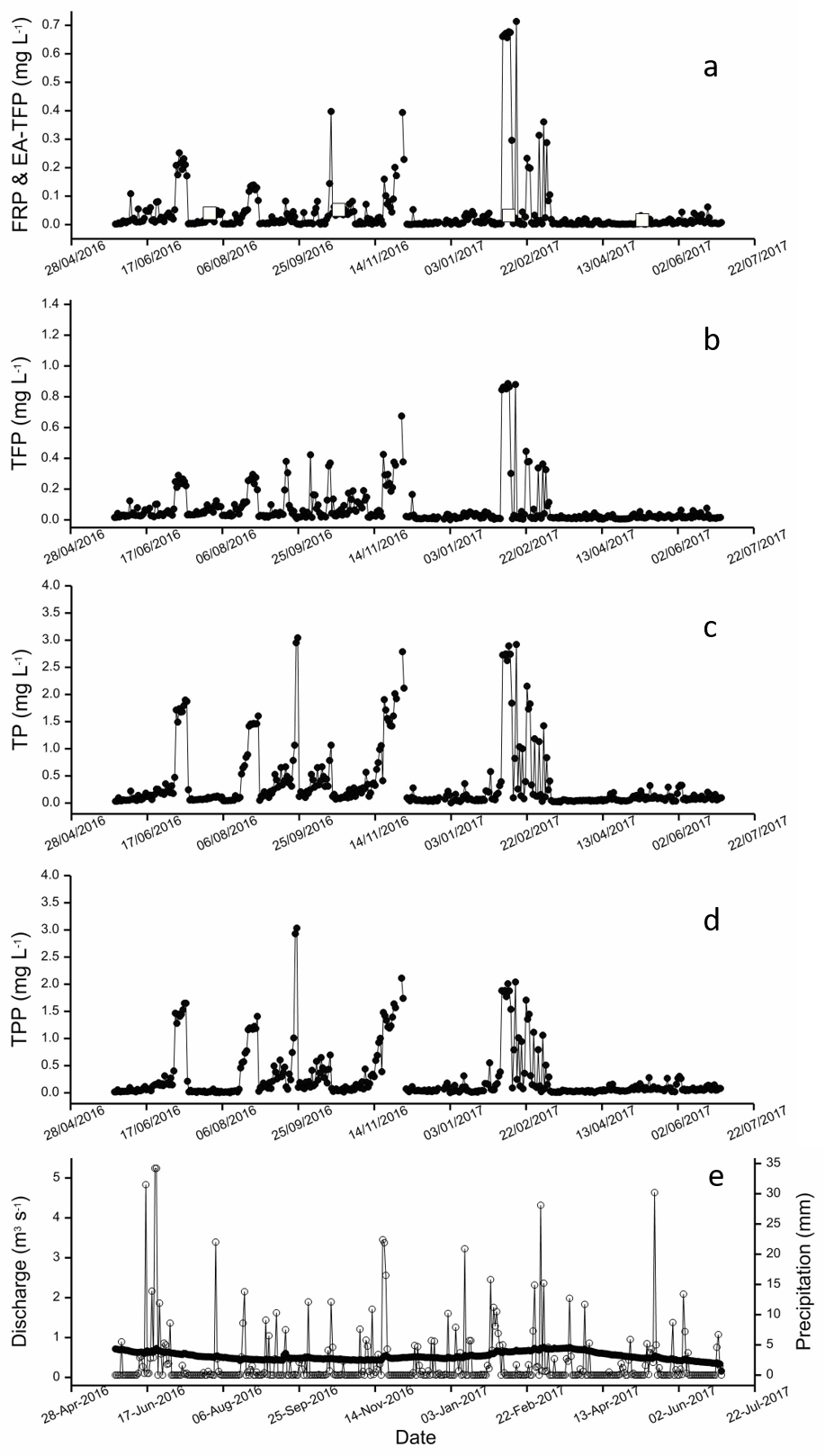
**

**Figure S8.** Variation in the concentration of phosphorus (mg L^-1^) fractions (•) in water samples collected (27 May 2016 – 30 June 2017) at site ABS 2 on the upper River Itchen (a) filterable reactive phosphorous (FRP); (b) total filterable phosphorous (TFP); (c) total phosphorous (TP); (d) total particulate phosphorous (TPP). Precipitation (mm) (⭘) and river discharge (m^3^ s^-1^) (•) measured at gauging station GS 1 over this period is shown (e). The concentration of total reactive phosphorous (mg L^-1^) (EA-TRP) () measured by the Environment Agency at EA 2 (River Arle at Drove Lane) is shown in (a).

**
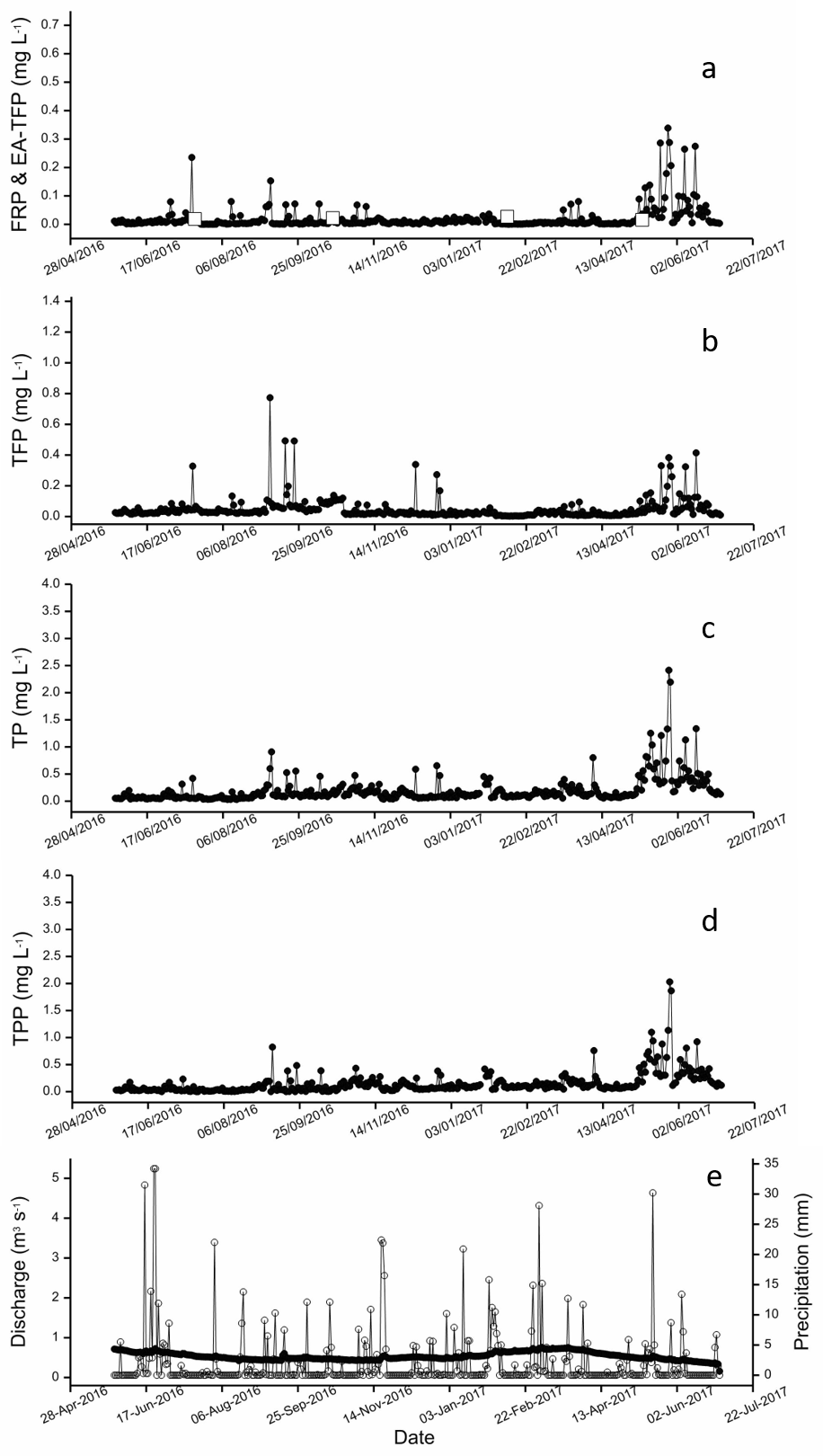
**

**Figure S9.** Variation in the concentration of phosphorus (mg L^-1^) fractions (•) in water samples collected (27 May 2016 – 30 June 2017) at site ABS 4 on the upper River Itchen (a) filterable reactive phosphorous (FRP); (b) total filterable phosphorous (TFP); (c) total phosphorous (TP); (d) total particulate phosphorous (TPP). Precipitation (mm) (⭘) and river discharge (m^3^ s^-1^) (•) measured at gauging station GS 1 over this period is shown (e). The concentration of total reactive phosphorous (mg L^-1^) (EA-TRP) () measured by the Environment Agency at EA 3 (River Itchen at Itchen Stoke) is shown in (a).

**
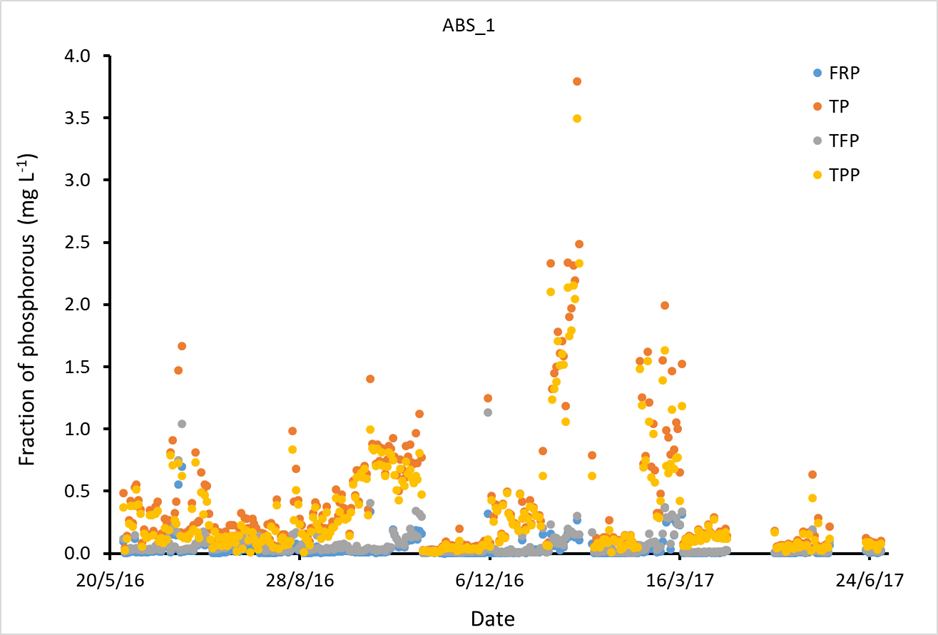
**

**Figure S10:** Variation in the concentration of phosphorous (mg L^-1^) fractions collected using the automated bottle sampler at site ABS 1 over the deployment period (27 May 2016 – 30 June 2017). FRP = filterable reactive phosphorous; TFP = total filterable phosphorous; TP = total phosphorous; TPP = total particulate phosphorous.


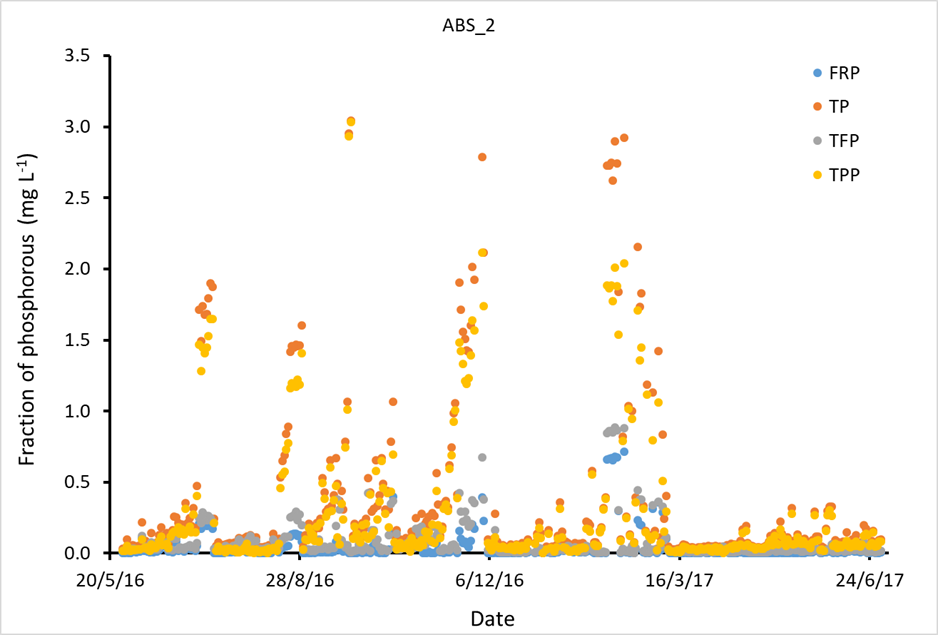


**Figure S11:** Variation in the concentration of phosphorous (mg L^-1^) fractions collected using the automated bottle sampler at site ABS 2 over the deployment period (27 May 2016 – 30 June 2017). FRP = filterable reactive phosphorous; TFP = total filterable phosphorous; TP = total phosphorous; TPP = total particulate phosphorous.


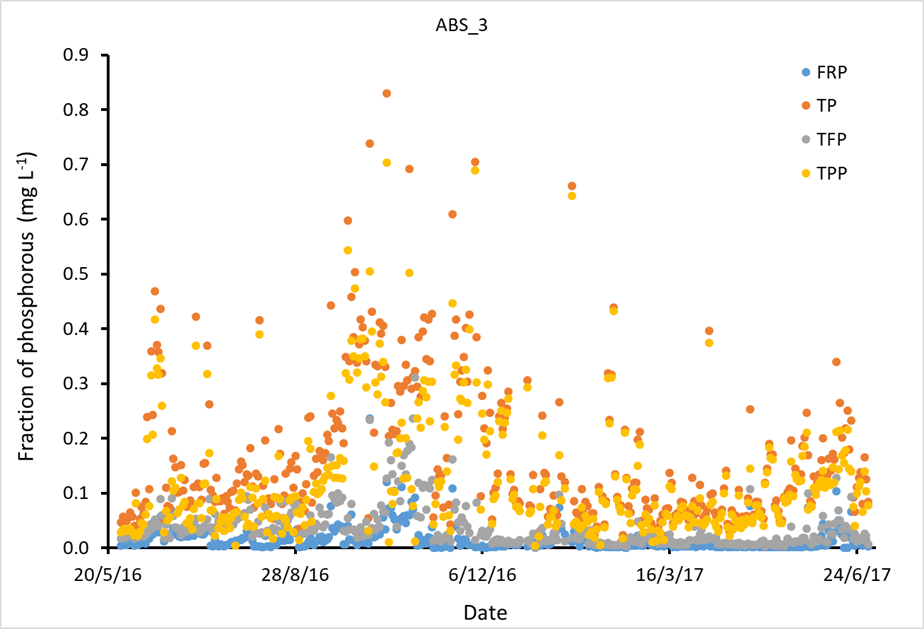


**Figure S12:** Variation in the concentration of phosphorous (mg L^-1^) fractions collected using the automated bottle sampler at site ABS 3 over the deployment period (27 May 2016 – 30 June 2017). FRP = filterable reactive phosphorous; TFP = total filterable phosphorous; TP = total phosphorous; TPP = total particulate phosphorous.


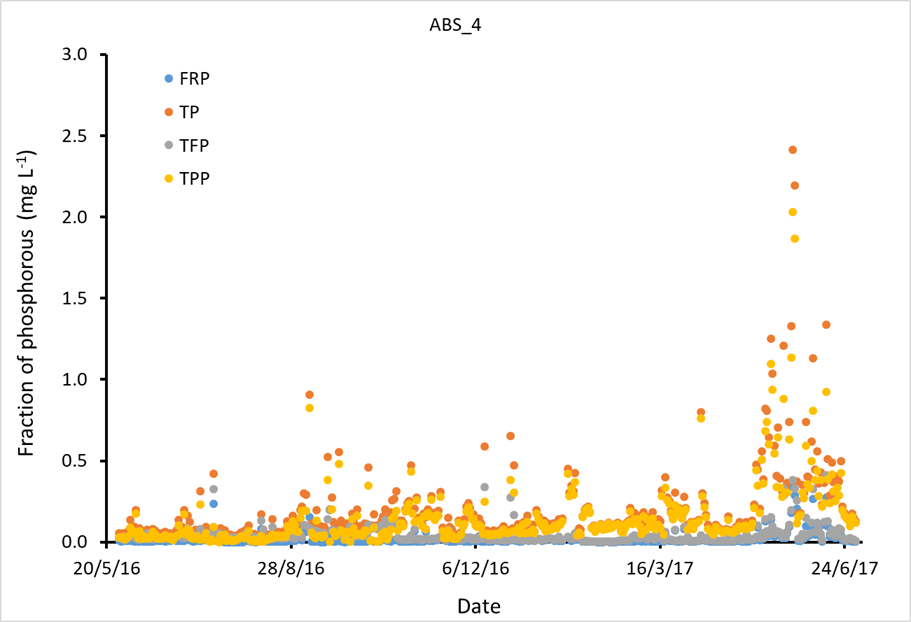


**Figure S13:** Variation in the concentration of phosphorous (mg L^-1^) fractions collected using the automated bottle sampler at site ABS 4 over the deployment period (27 May 2016 – 30 June 2017). FRP = filterable reactive phosphorous; TFP = total filterable phosphorous; TP = total phosphorous; TPP = total particulate phosphorous.


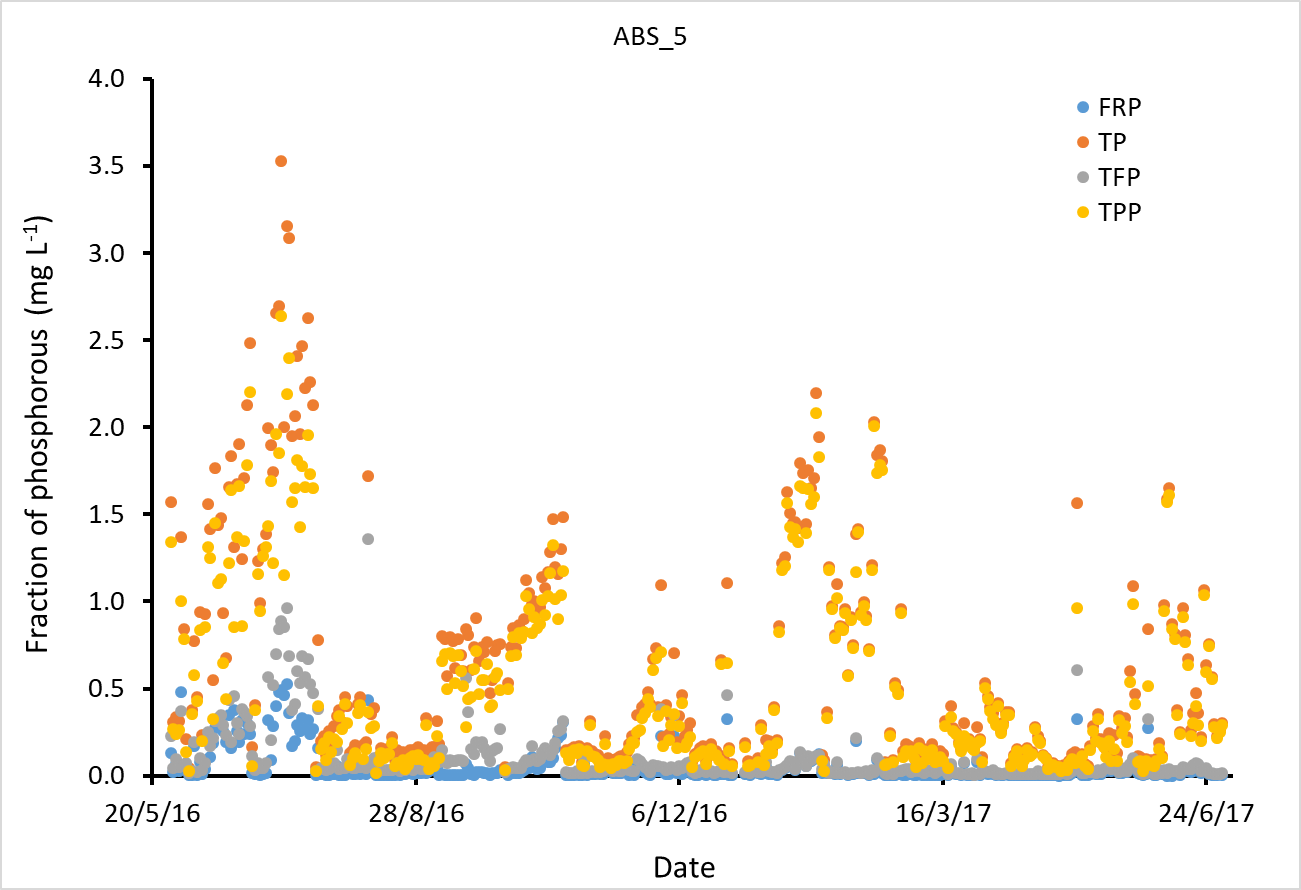


**Figure S14:** Variation in the concentration of phosphorous (mg L^-1^) fractions collected using the automated bottle sampler at site ABS 5 over the deployment period (27 May 2016 – 30 June 2017). FRP = filterable reactive phosphorous; TFP = total filterable phosphorous; TP = total phosphorous; TPP = total particulate phosphorous.

**
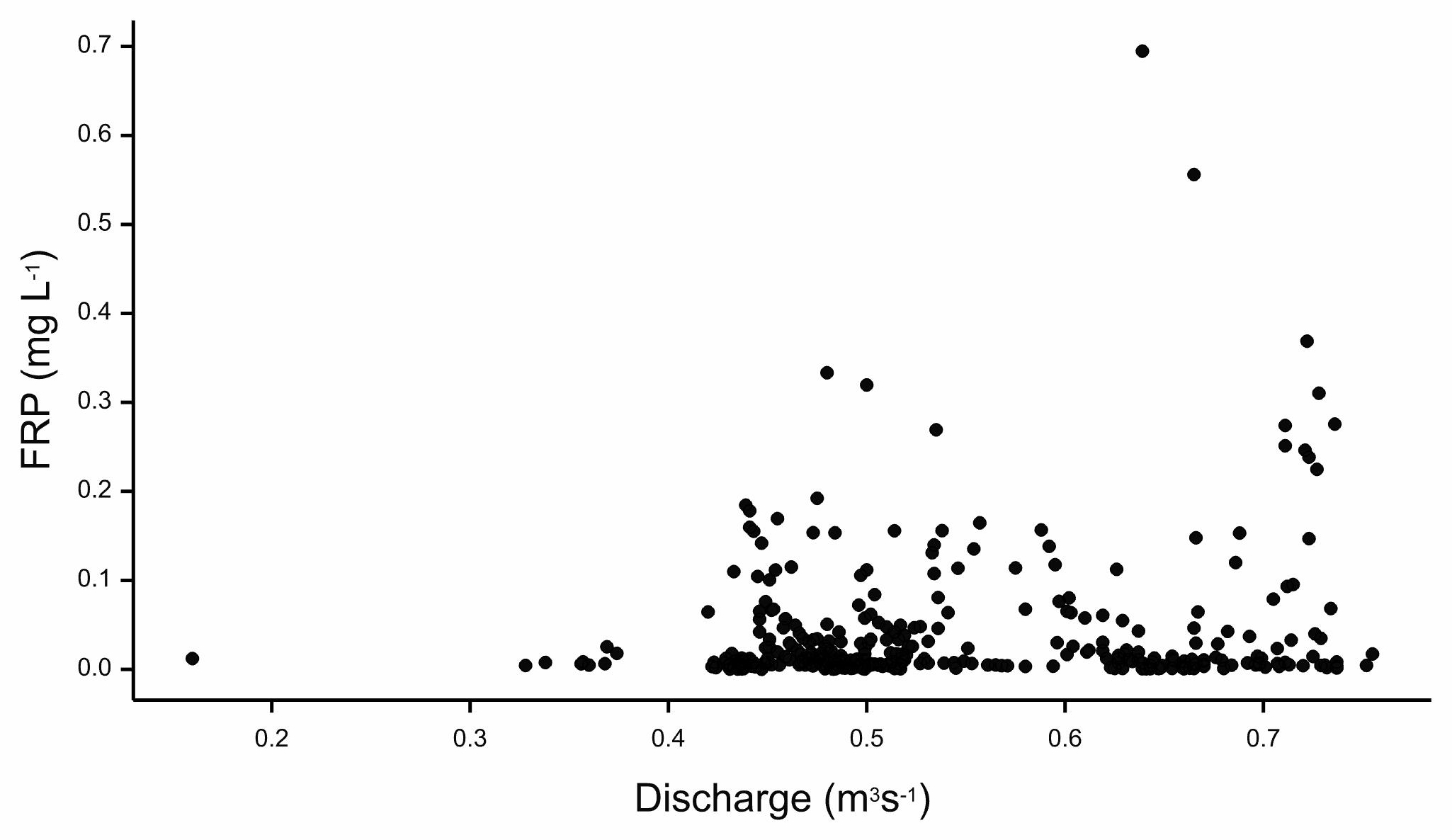
Figure S15:** Variation in the concentration of filterable reactive phosphorous (FRP) (mg L^-1^) with river discharge (m^3^ s^-1^) at site ABS 1 over the deployment period (27 May 2016 – 30 June 2017). River discharge data obtained from gauging station 1 (Candover Stream at Borough Bridge: 42009).

**
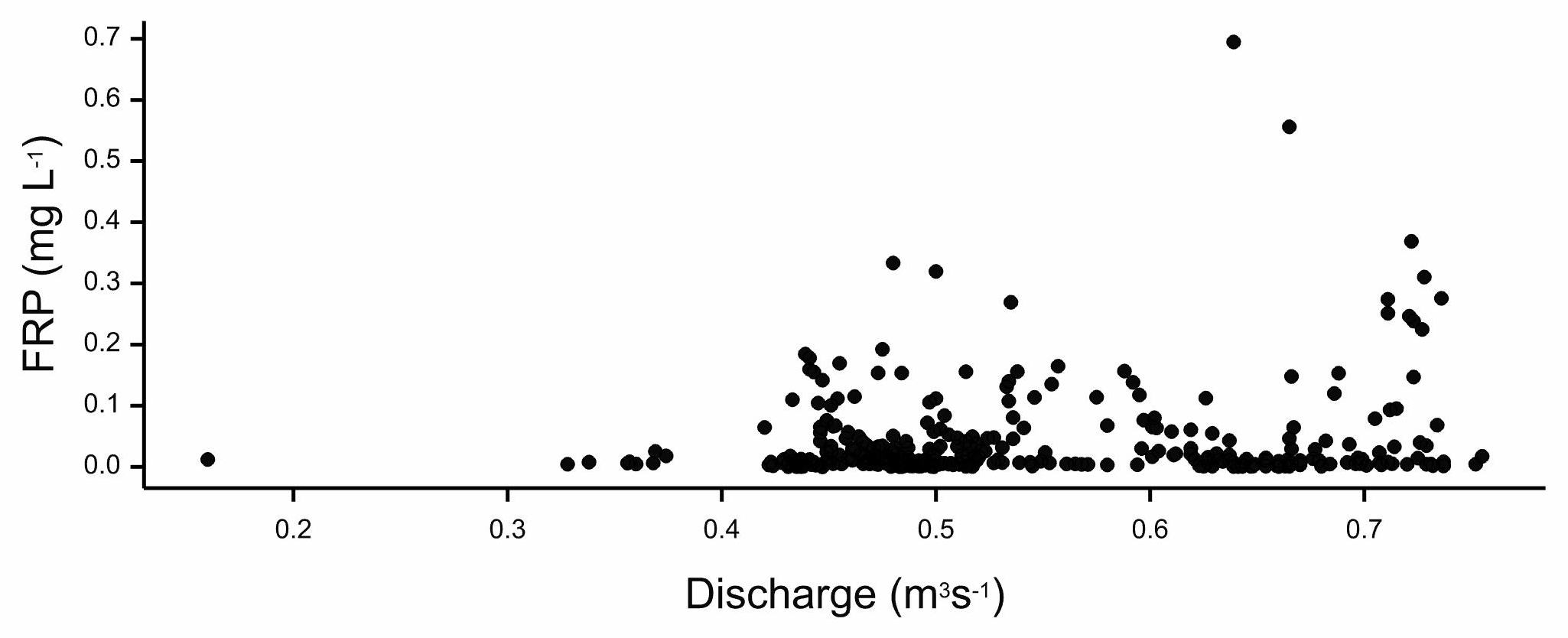
**

**Figure S16:** Variation in the concentration of filterable reactive phosphorous (FRP) (mg L^-1^) with river discharge (m^3^ s^-1^) at site ABS 2 over the deployment period (27 May 2016 – 30 June 2017). River discharge data obtained from gauging station 1 (Candover Stream at Borough Bridge: 42009).

**
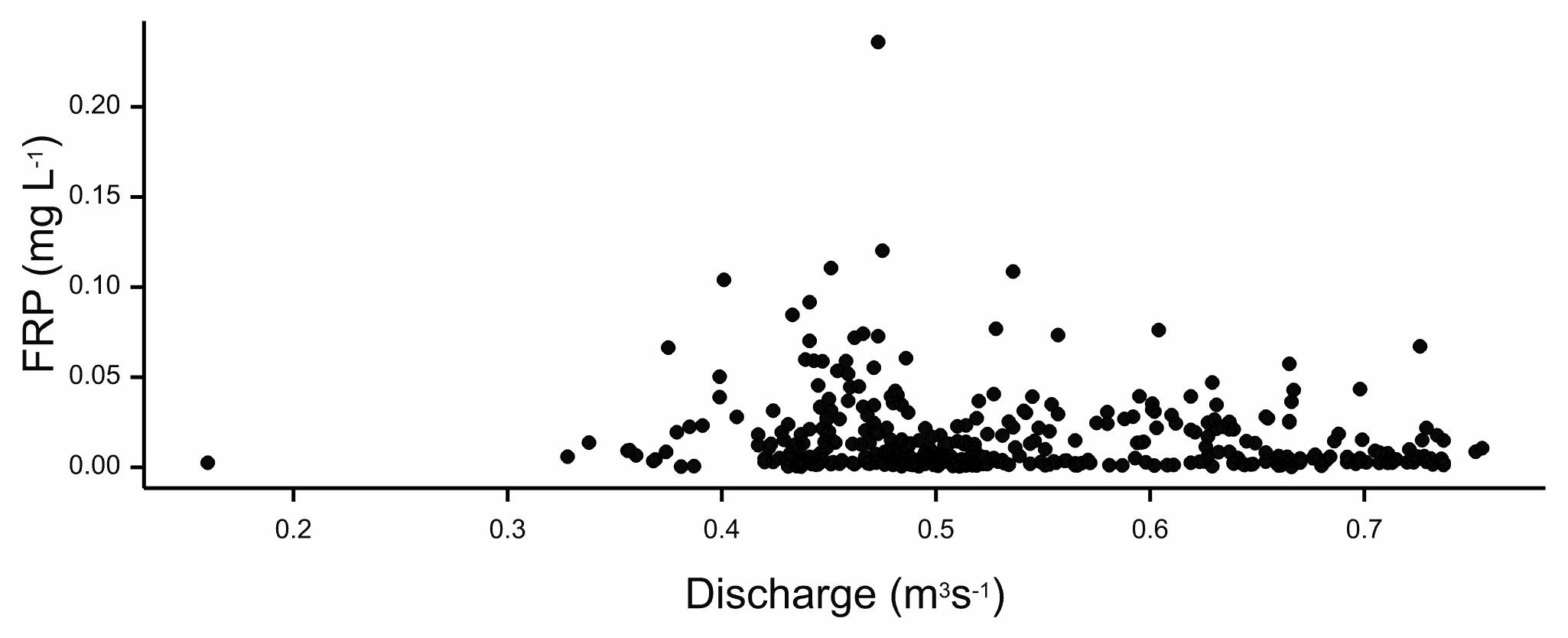
**

**Figure S17:** Variation in the concentration of filterable reactive phosphorous (FRP) (mg L^-1^) with river discharge (m^3^ s^-1^) at site ABS 3 over the deployment period (27 May 2016 – 30 June 2017). River discharge data obtained from gauging station 1 (Candover Stream at Borough Bridge: 42009).

**
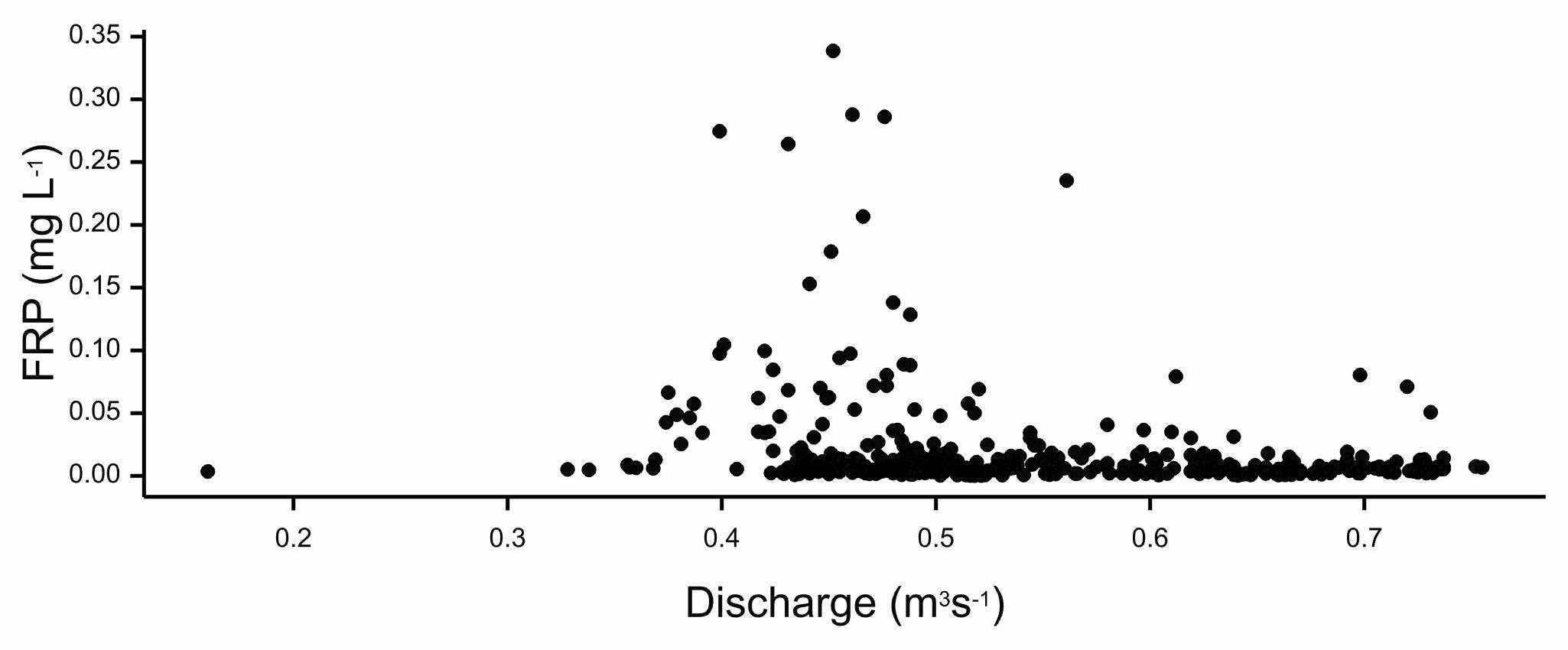
**

**Figure S18:** Variation in the concentration of filterable reactive phosphorous (FRP) (mg L^-1^) with river discharge (m^3^ s^-1^) at site ABS 4 over the deployment period (27 May 2016 – 30 June 2017). River discharge data obtained from gauging station 1 (Candover Stream at Borough Bridge: 42009).

**
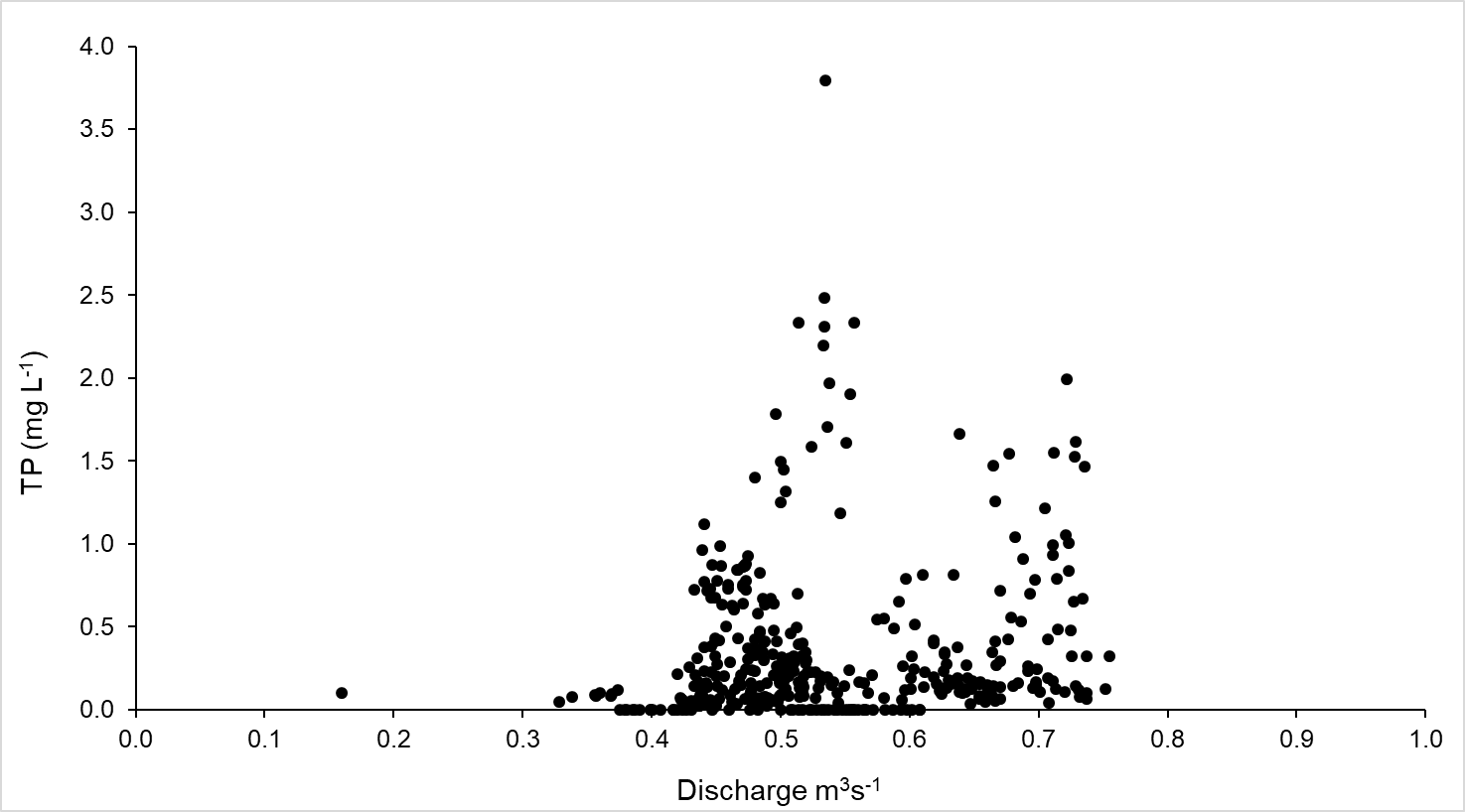
**

**Figure S19:** Variation in the concentration of total phosphorous (TP) (mg L^-1^) with river discharge (m^3^ s^-1^) at site ABS 1 over the deployment period (27 May 2016 – 30 June 2017). River discharge data obtained from gauging station 1 (Candover Stream at Borough Bridge: 42009).

**
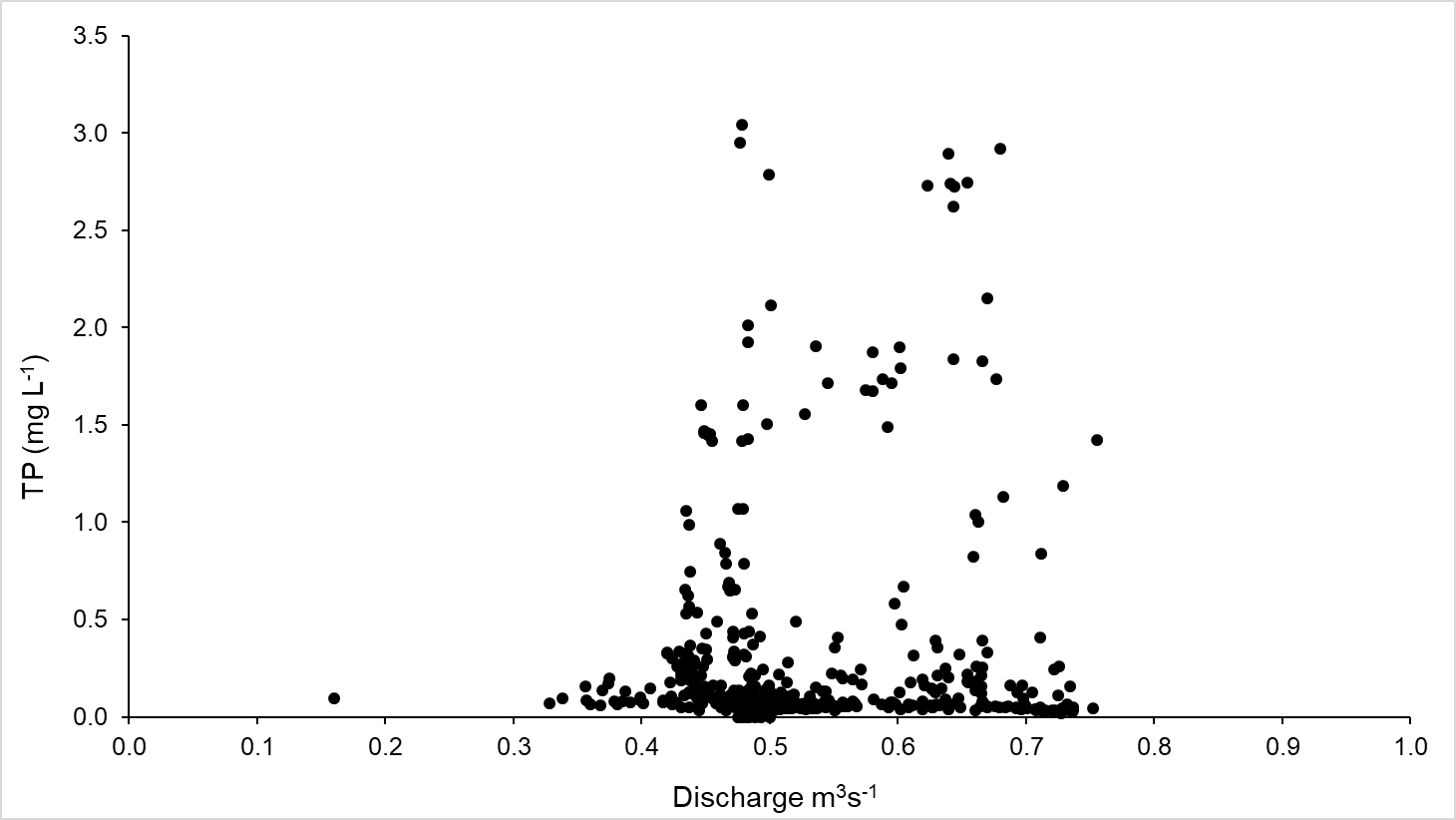
**

**Figure S20:** Variation in the concentration of total phosphorous (TP) (mg L^-1^) with river discharge (m^3^ s^-1^) at site ABS 2 over the deployment period (27 May 2016 – 30 June 2017). River discharge data obtained from gauging station 1 (Candover Stream at Borough Bridge: 42009).

**
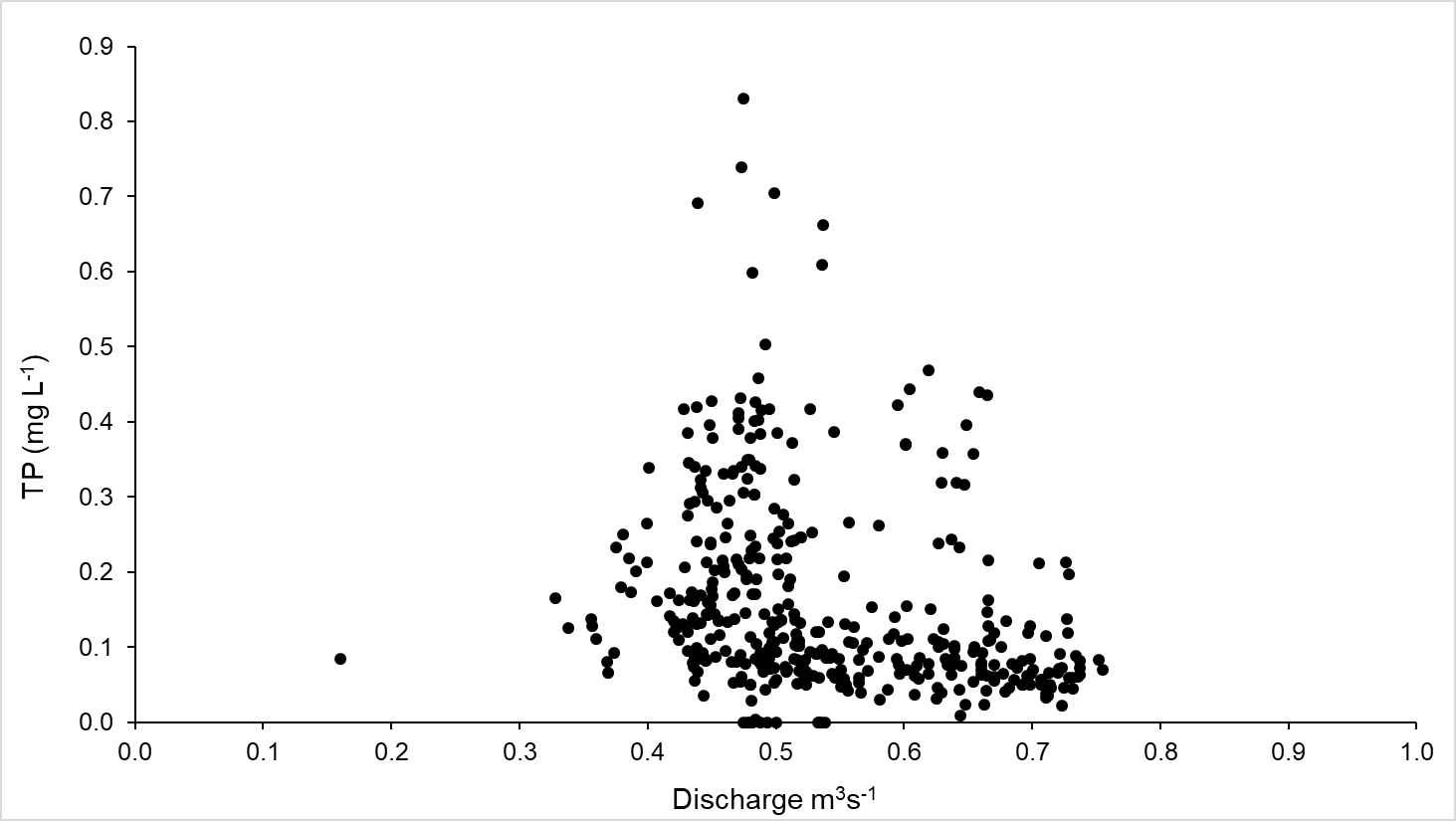
**

**Figure S21:** Variation in the concentration of total phosphorous (TP) (mg L^-1^) with river discharge (m^3^ s^-1^) at site ABS 3 over the deployment period (27 May 2016 – 30 June 2017). River discharge data obtained from gauging station 1 (Candover Stream at Borough Bridge: 42009).

**
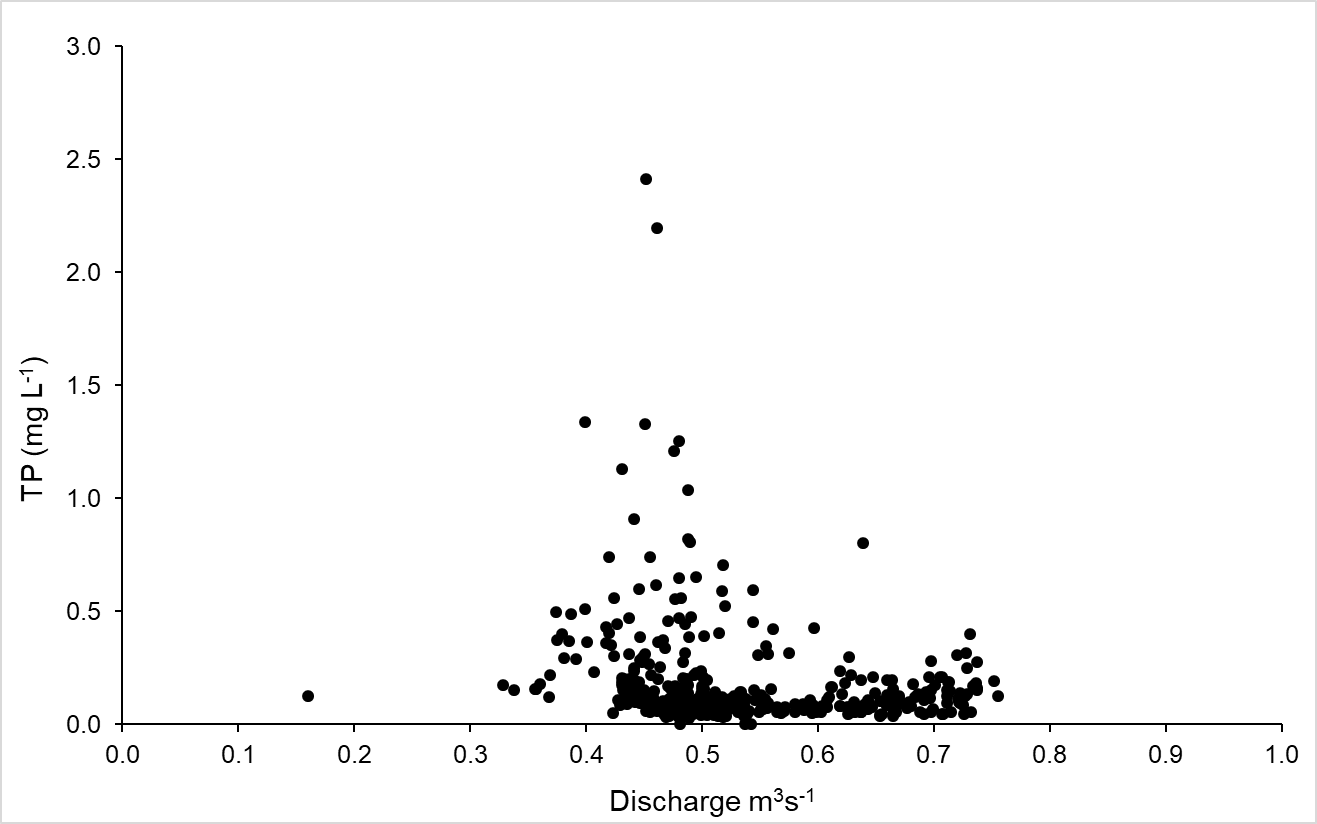
**

**Figure S22:** Variation in the concentration of total phosphorous (TP) (mg L^-1^) with river discharge (m^3^ s^-1^) at site ABS 4 over the deployment period (27 May 2016 – 30 June 2017). River discharge data obtained from gauging station 1 (Candover Stream at Borough Bridge: 42009).

**
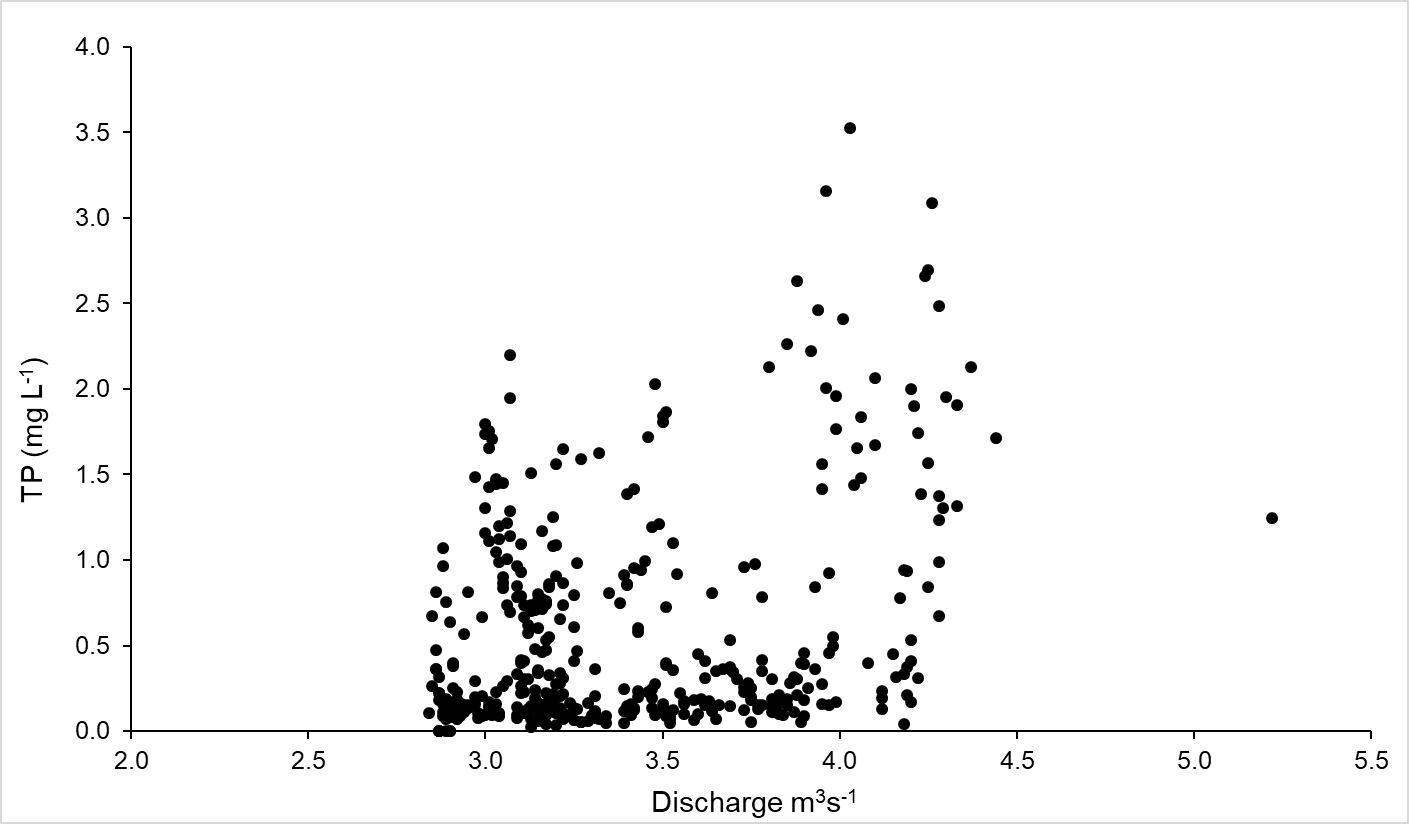
**

**Figure S23:** Variation in the concentration of total phosphorus (TP) (mg L^-1^) with river discharge (m^3^ s^-1^) at site ABS 5 over the deployment period (27 May 2016 – 30 June 2017). River discharge data obtained from gauging station 2 (Itchen at Easton: 42016).

**
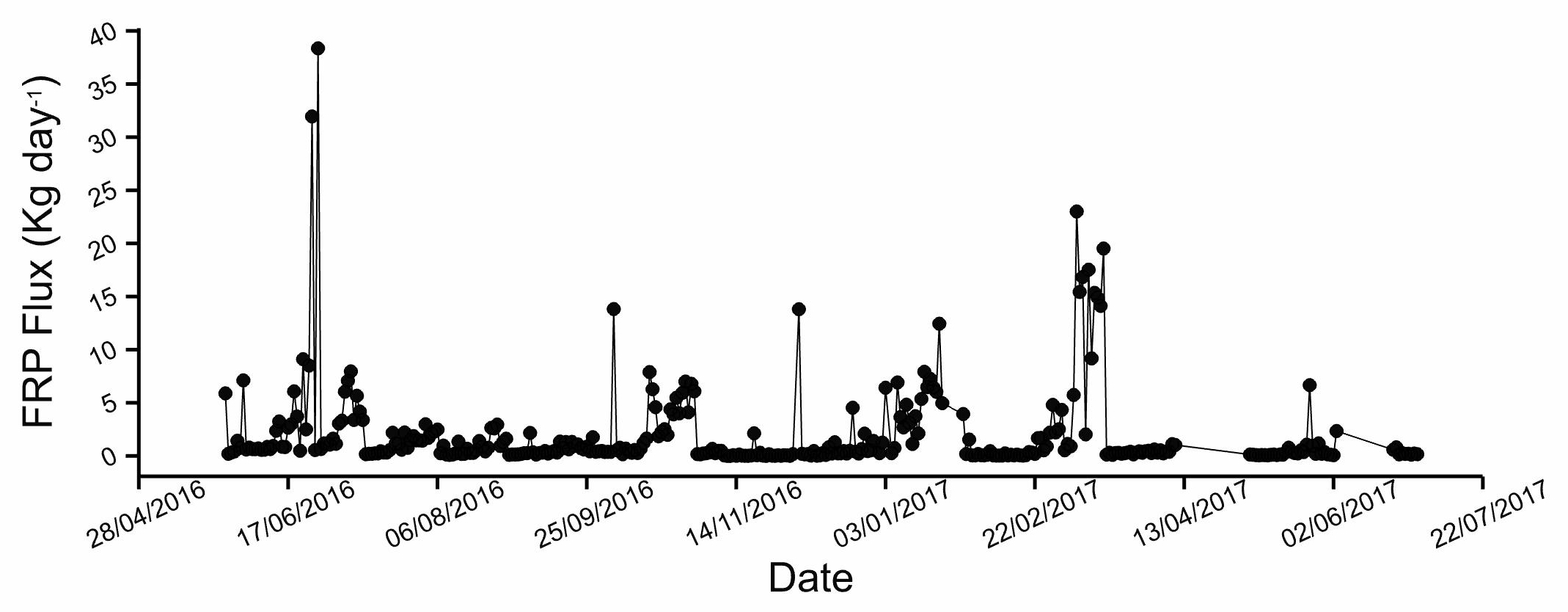
**

**Figure S24**: Variation in the flux (kg day^-1^) of filterable reactive phosphorous (FRP) at site ABS 1 over the deployment period (27 May 2016 – 30 June 2017). River discharge data obtained from gauging station 1 (Candover Stream at Borough Bridge: 42009).

**
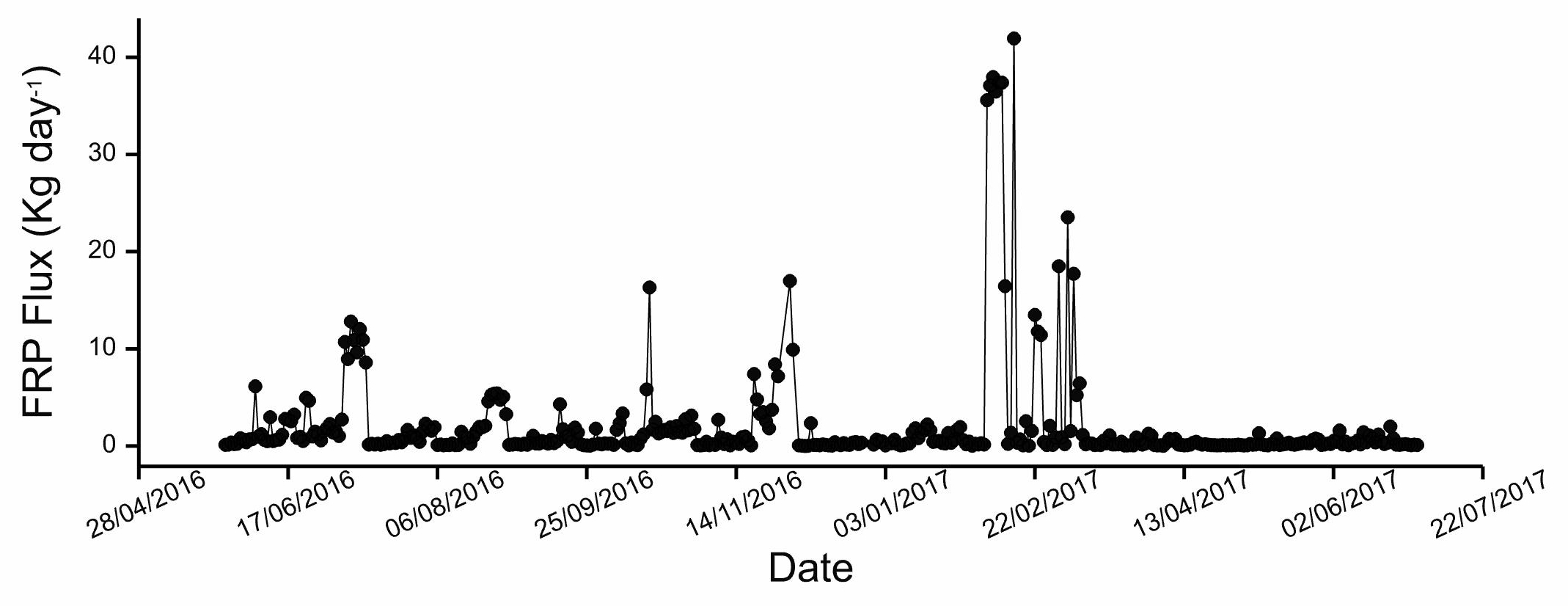
**

**Figure S25**: Variation in the flux (kg day^-1^) of filterable reactive phosphorous (FRP) at site ABS 2 over the deployment period (27 May 2016 – 30 June 2017). River discharge data obtained from gauging station 1 (Candover Stream at Borough Bridge: 42009).


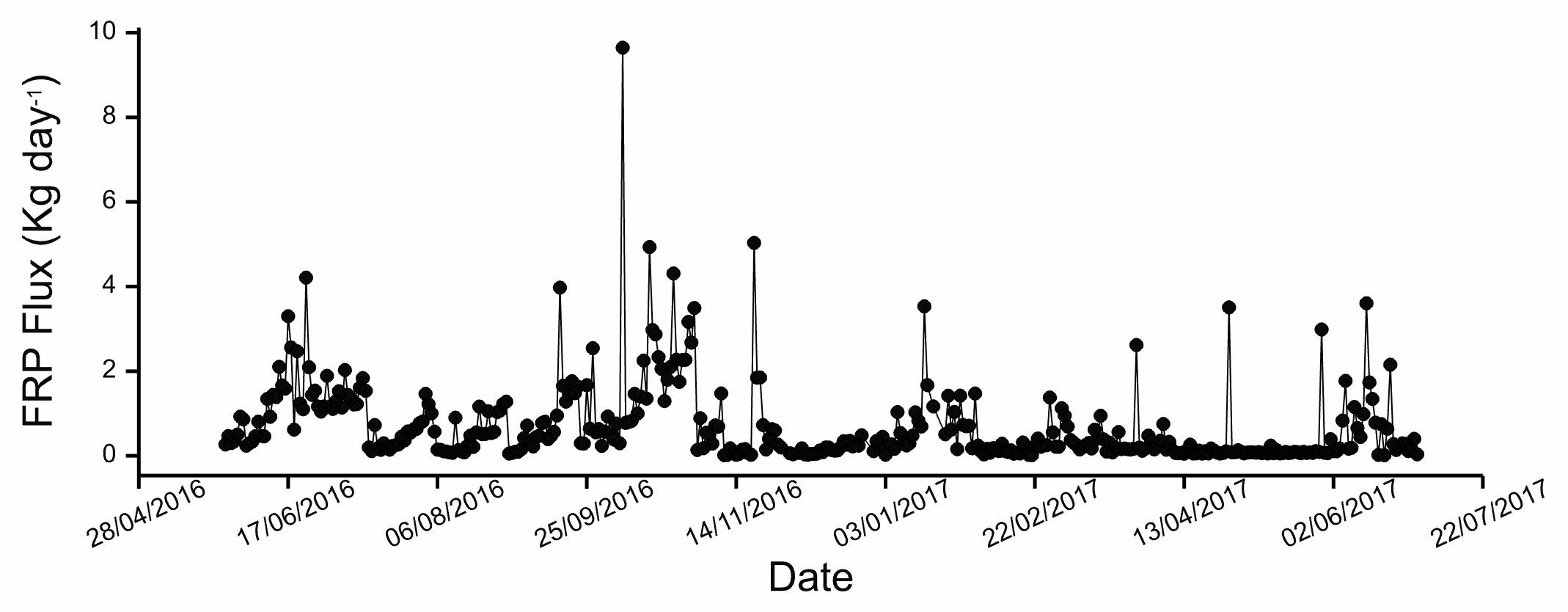


**Figure S26**: Variation in the flux (kg day^-1^) of filterable reactive phosphorous (FRP) at site ABS 3 over the deployment period (27 May 2016 – 30 June 2017). River discharge data obtained from gauging station 1 (Candover Stream at Borough Bridge: 42009).


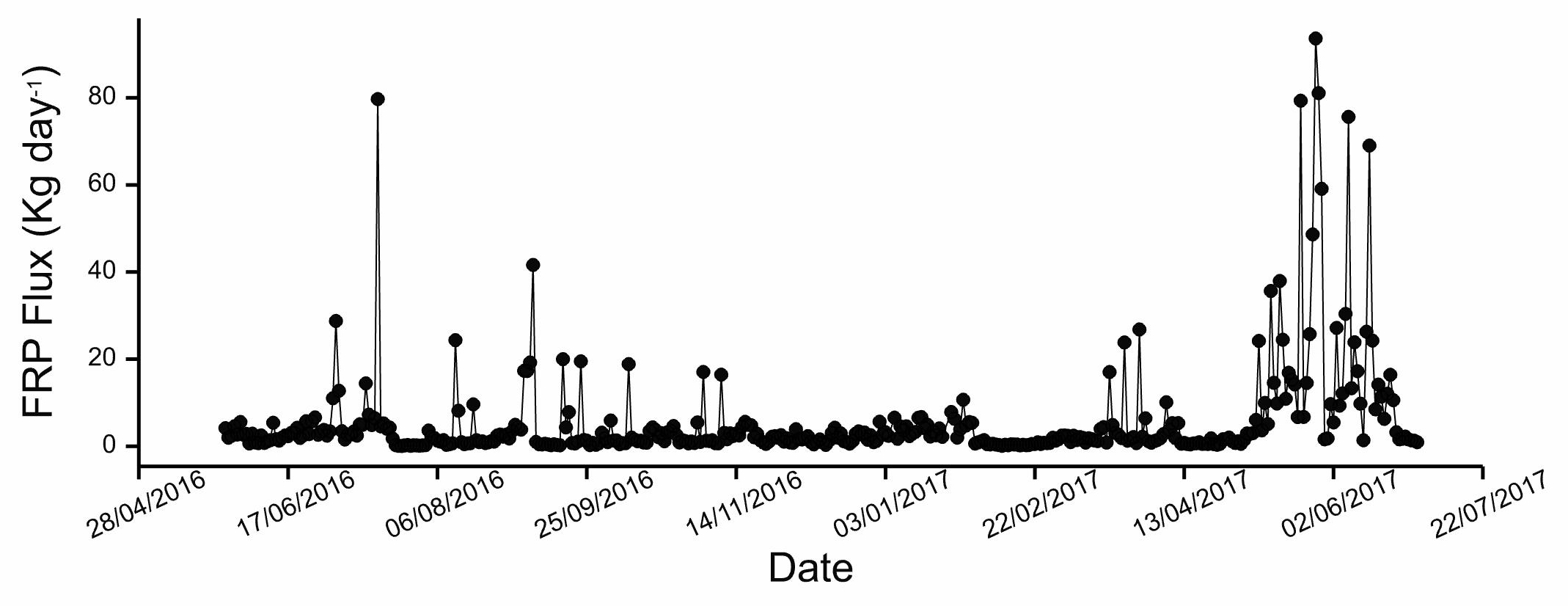


**Figure S27**: Variation in the flux (kg day^-1^) of filterable reactive phosphorous (FRP) at site ABS 4 over the deployment period (27 May 2016 – 30 June 2017). River discharge data obtained from gauging station 1 (Candover Stream at Borough Bridge: 42009).


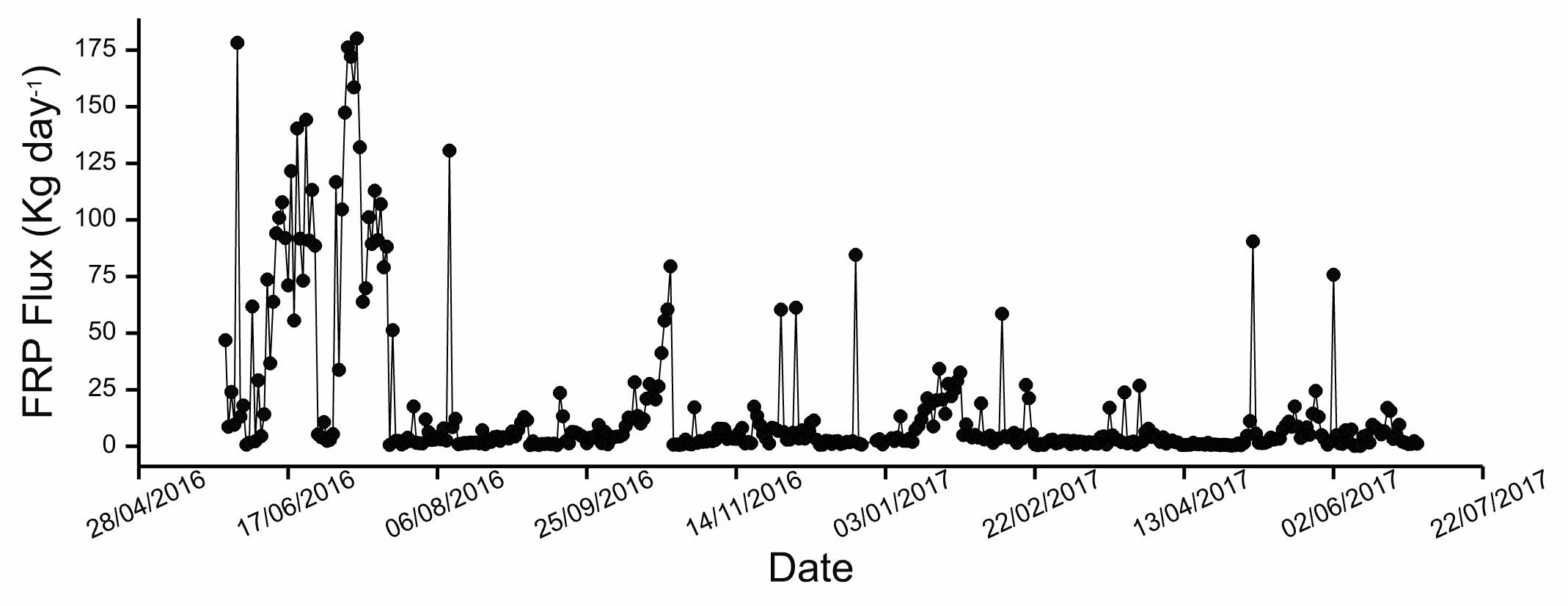


**Figure S28**: Variation in the flux (kg day^-1^) of filterable reactive phosphorous (FRP) at site ABS 5 over the deployment period (27 May 2016 – 30 June 2017). River discharge data obtained from gauging station 2 (Itchen at Easton: 42016).

**Table S2**: UK Technical Advisory Group on the Water Framework Directive standards for the mean concentration (mg L^-1^) of phosphorous in rivers. Data based on 819 differing sites (The numbers in parentheses are the upper and lower 5^th^ and 95^th^ percentiles of the standards for the sites in each type).

| **River Type** | **Annual mean of reactive phosphorous (mg L^-1^)** | | | |
| --- | --- | --- | --- | --- |
|  | **High** | **Good** | **Moderate** | **Poor** |
| **High altitude, low alkalinity** | 0.013  (0.013-0.020) | 0.028  (0.028-0.041) | 0.087  (0.087-0.117) | 0.752  (0.752-0.918) |
| **High altitude, high alkalinity** | 0.024  (0.018-0.037) | 0.048  (0.028-0.070) | 0.132  (0.109-0.177) | 0.898  (0.829-1.012) |
| **Low altitude, low alkalinity** | 0.019  (0.013-0.026) | 0.040  (0.028-0.052) | 0.114  (0.087-0.140) | 0.842  (0.752-0.918) |
| **Low altitude, high alkalinity** | 0.036  (0.027-0.050) | 0.069  (0.052-0.091) | 0.173  (0.141-0.215) | 1.003  (0.921-1.098) |

Notes: High altitude > 80 m, high alkalinity > 50 mg L^-1^ CaCO_3_

**Table S3**: Common Standards Monitoring Guidance for Rivers (January 2014) proposed phosphorus targets (mg L^-1^) consistent with favourable condition of SSSI/SAC riverine habitat.

| **River Type** | **Headwater** | **River** | **Large river** |
| --- | --- | --- | --- |
| **High altitude, low alkalinity** | 0.010 | 0.020 | 0.030 |
| **High altitude, high alkalinity** | 0.015 | 0.025 | 0.040 |
| **Low altitude, low alkalinity** | 0.030 | 0.040 | 0.050 |
| **Low altitude, high alkalinity** | 0.040 | 0.050 | 0.050 |

Notes: High altitude > 80 m, high alkalinity > 50 mg L^-1^ CaCO_3_
